# Extracellular vesicle-packaged GBP2 from macrophages aggravates sepsis-induced acute lung injury by promoting ferroptosis in pulmonary vascular endothelial cells

**DOI:** 10.1016/j.redox.2025.103614

**Published:** 2025-03-25

**Authors:** Zhixi Li, Yue Bu, Cheng Wang, Yongjing Yu, Lei Han, Chang Liu, Guangmin Chen, Chenglong Li, Yan Zhang, Hang Cao, Zhaoxue Ma, Ziyong Yue

**Affiliations:** a Department of Anesthesiology, Second Affiliated Hospital of Harbin Medical University, Harbin, 150001, PR China; b The Key Laboratory of Anesthesiology and Intensive Care Research of Heilongjiang Province, Harbin, 150001, PR China; c The Key Laboratory of Myocardial Ischemia Organization, Chinese Ministry of Education, Harbin, 150001, PR China; d State Key Laboratory of Frigid Zone Cardiovascular Diseases, Harbin, 150001, PR China; e Department of Pain Medicine, Second Affiliated Hospital of Harbin Medical University, Harbin, 150001, PR China; f Department of Environmental Hygiene, School of Public Health, Harbin Medical University, Harbin, 150081, PR China; g Department of Anesthesiology, Harbin Medical University Cancer Hospital, Harbin, 150081, PR China; h Department of Anesthesiology, First Affiliated Hospital of Harbin Medical University, 199 Dazhi Road, Harbin, 150001, PR China; i Department of Anesthesiology, Fourth Affiliated Hospital of Harbin Medical University, 37 Yiyuan Road, Harbin, 150001, PR China

**Keywords:** Sepsis, Acute lung injury, Macrophage, Endothelial cells, Extracellular vesicles, Ferroptosis

## Abstract

Macrophages play a critical role in the development of sepsis-induced acute lung injury (si-ALI), with extracellular vesicles (EVs) acting as crucial mediators. However, the effects and mechanisms of macrophage-derived EVs on si-ALI remain unclear. This study demonstrated that macrophage-derived EVs induce endothelial ferroptosis and barrier disruption during sepsis. Through proteomic sequencing and reanalysis of transcriptomic and single-cell sequencing data, guanylate-binding protein 2 (GBP2) was identified as a key EV molecule. Elevated GBP2 expression was observed in EVs and monocytes from the peripheral blood of sepsis patients, in LPS-stimulated THP-1 and RAW264.7 cells and their secreted EVs, and in macrophages within the lungs of CLP mice. Additionally, GBP2 expression in EVs showed a positive correlation with vascular barrier injury biomarkers, including ANGPT2, Syndecan-1, and sTM. Modulating GBP2 levels in macrophage-derived EVs affected EV-induced ferroptosis in endothelial cells. The mechanism by which GBP2 binds directly to OTUD5 and promotes GPX4 ubiquitination was elucidated using RNA interference, adeno-associated virus transfection, and endothelial-specific Gpx4 knockout mice. A high-throughput screening of small-molecule compounds targeting GBP2 was conducted. Molecular docking, molecular dynamics simulations, and cellular thermal shift assays further confirmed that Plantainoside D (PD) has a potent binding affinity for GBP2. PD treatment inhibited the interaction between GBP2 and OTUD5, leading to a reduction in GPX4 ubiquitination. Further research revealed that PD treatment enhanced the pulmonary protective effects of GBP2 inhibition. In conclusion, this study explored the role of EV-mediated signaling between macrophages and pulmonary vascular endothelial cells in si-ALI, highlighting the GBP2-OTUD5-GPX4 axis as a driver of endothelial ferroptosis and lung injury. Targeting this signaling axis presents a potential therapeutic strategy for si-ALI.

## Highlights

• During sepsis-induced acute lung injury, GBP2 is enriched in macrophage and macrophage-derived extracellular vesicles.
• Macrophage-derived EV GBP2 is transferred to endothelial cells, where it interacts with OTUD5 to promote GPX4 ubiquitination.
• Plantainoside D binds GBP2 and alleviates EV-induced endothelial ferroptosis by modulating the GBP2-OTUD5-GPX4 axis.

## Graphical abstract

**Figure:**
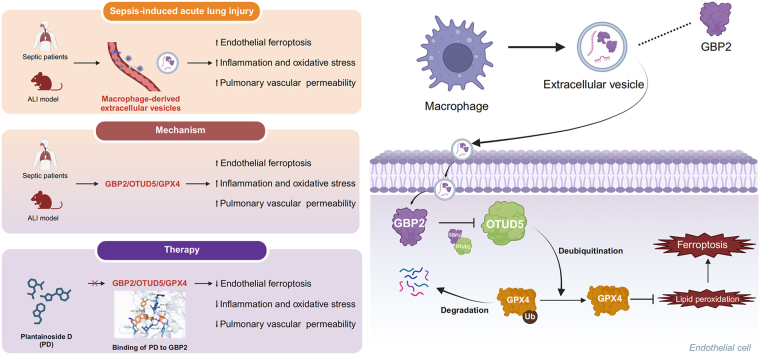

## 1 Introduction

Sepsis is a systemic inflammatory response syndrome resulting from pathogenic microorganisms' infection, leading to organ dysfunction [1]. Sepsis-induced acute lung injury (si-ALI) is a common complication of sepsis, often triggered by pulmonary or extrapulmonary infections, and is characterized by lung damage caused by excessive inflammatory responses or concurrent bacterial infection. In contrast to secondary bacterial pneumonia, which primarily involves direct bacterial colonization and infection of the lungs, si-ALI is frequently associated with a dysregulated systemic inflammatory response and is frequently correlated with a worse prognosis [2]. Although recent research has advanced the understanding of si-ALI and improved treatment strategies, patient outcomes remain suboptimal, and mortality rates remain high [3]. Further investigation into the pathological mechanisms of si-ALI is crucial for identifying effective therapeutic targets and improving patient prognosis.

The disruption of the pulmonary microvascular endothelial barrier is a key pathological event during the acute phase of si-ALI, leading to alveolar edema and pulmonary dysfunction [4]. Therefore, maintaining endothelial cell homeostasis and restoring the integrity of the endothelial barrier are critical for si-ALI treatment. Ferroptosis is a form of programmed cell death induced by iron overload and lipid peroxidation [5,6]. Recent studies suggest that endothelial cells are highly sensitive to ferroptosis, and inhibiting ferroptosis may improve the prognosis of sepsis [6,7]. Glutathione peroxidase 4 (GPX4), a phospholipid peroxidase, maintains redox balance by inhibiting lipid peroxidation, thereby preventing the onset and progression of ferroptosis [8]. Research suggests that disruption of GPX4 homeostasis and reduced GPX4 expression enhance ferroptosis sensitivity [8]. However, the precise molecular mechanisms regulating ferroptosis in sepsis remain poorly understood.

Macrophages play a crucial role in sepsis progression. Our previous research revealed that macrophage infiltration increases in the early stages of sepsis, substantially contributing to tissue damage [9]. However, the precise mechanisms by which macrophages drive this damage remain incompletely understood and warrant further investigation. Extracellular vesicles (EVs) have been implicated in sepsis progression by regulating biological processes, such as inflammation, oxidative stress, and cell death [10,11]. Recent studies indicate that macrophages influence the function of other cells through EV pathways [12]. Furthermore, macrophage-derived EVs have been shown to impair mitochondrial function in vascular endothelial cells and inhibit angiogenesis [13,14], underscoring EV-mediated signaling as a potential communication mechanism between macrophages and endothelial cells. However, the precise role and underlying mechanisms of macrophage-derived EVs in regulating pulmonary vascular endothelial cells during si-ALI remain unclear.

This study investigated the effects and mechanisms of macrophage-derived EVs on pulmonary microvascular endothelial cells and lung tissues in sepsis. Our findings demonstrate that during si-ALI, macrophages induce endothelial ferroptosis and barrier disruption through EV pathways. Using proteomics, transcriptomics, single-cell sequencing, clinical sample analysis, and further experimental validation, guanylate-binding protein 2 (GBP2) was identified as a key molecule in this process. Mechanistically, GBP2 interacts with OTU deubiquitinase 5 (OTUD5) in endothelial cells, promoting the ubiquitination of GPX4, thereby increasing sensitivity to ferroptosis and compromising the endothelial barrier. Moreover, Plantainoside D (PD) was screened as a compound that binds effectively to GBP2, with PD treatment enhancing lung protection in combination with GBP2 inhibition. This study elucidates a novel pathological mechanism of pulmonary vascular injury during si-ALI and highlights the potential of targeting GBP2 as a promising therapeutic approach to mitigate si-ALI.

## 2 Materials and methods

### 2.1 Reagent

Mouse Angpt2, IL-1β, IL-6, and TNF-α, and human GBP2, ANGPT2, CRP, Syndecan-1, and sTM ELISA kits were purchased from Jingkang (Shanghai, China). Plantainoside D (HY–N5063), Ferrostatin-1 (HY-100579), and GW4869 (HY-19363) were obtained from MedChemExpress (NJ, USA). LPS (L2630), PMA (P1585), and PKH67 were purchased from Sigma-Aldrich (MO, USA). Lipofectamine™ 2000 was obtained from Invitrogen (MD, USA). DiR was purchased from Umibio (Shanghai, China). Phalloidin (MX4405) was obtained from Mkbio (Shanghai, China). PI, DAPI, Hoechst, JC-1, DHE, and ROS staining kits were purchased from Beyotime (Shanghai, China). Zombie NIR™ dye was obtained from BioLegend (CA, USA). CCK8 and liperfluo were provided by DOJINDO (Kumamoto, Japan). AceQ qPCR SYBR Green Master Mix kit (Q111-03) was supplied by Vazyme (Nanjing, China). The first Strand cDNA Synthesis Kit (FSK-101) was obtained from TOYOBO (Osaka, Japan).

### 2.2 Animal experiments

This study used male C57BL/6 mice weighing 20–25g, obtained from the Animal Experiment Center of the Second Affiliated Hospital of Harbin Medical University. Gpx4^f/f^ and Cdh5-cre mice, all on a C57BL/6 background, were obtained from Cyagen Biosciences. The experimental protocol was approved by the Ethics Committee (Approval No.: SYDW2021-087).

Construction of the CLP model:To establish the sepsis-induced acute lung injury (si-ALI) model, the cecal ligation and puncture (CLP) method was employed [15]. Mice were anesthetized with sevoflurane, and a midline incision was made to expose the cecum. The cecum was ligated at its midpoint with a 4-0 surgical suture and punctured with a 20 G needle. A small amount of feces was extruded to confirm the patency of the puncture site. The cecum was then returned to the abdominal cavity, and the incision was closed in layers. Mice received subcutaneous saline (50 μL/g) and were kept in a warm environment until recovery, then maintained under standard conditions. Sham-operated mice underwent the same procedure, excluding cecal ligation and puncture.

Intraperitoneal injection of LPS: Mice were intraperitoneally injected with 10 mg/kg of LPS or vehicle [16]. Twenty-four hours post-injection, the mice were euthanized for further sampling and analysis.

Intratracheal administration of EVs: A mouse macrophage-derived EVs (mEVs) suspension was prepared in PBS. The protein concentration of mEV suspensions was measured using the BCA assay [17]. After anesthetizing the mice, the mEV suspension (5 μg/g) was administered intratracheally. Subsequently, mice underwent either CLP modeling or sham operation. Twenty-four hours post-operation, mice were re-anesthetized for blood collection and euthanized for further sampling and analysis [18,19].

PD administration: The administration of PD was referenced in previous literature [20]. PD (30 mg/kg) (intravenous injection) was given to mice for five consecutive days. Twenty-four hours after CLP modeling, mice were anesthetized for blood collection, followed by euthanasia to facilitate further sampling and analysis.

### 2.3 Cell culture

THP-1 and RAW264.7 cell lines were obtained from the Cell Bank of the Chinese Academy of Sciences (Shanghai, China), while human pulmonary microvascular endothelial cells (HPMECs) were sourced from ScienCell. THP-1 cells were cultured in RPMI 1640 medium supplemented with 10 % FBS, and RAW264.7 cells were maintained in DMEM with 10 % FBS. HPMECs were cultured in the ECM medium supplemented with 5 % FBS and 1 % endothelial growth supplement. HPMECs were co-cultured with EVs (60 μg/mL) for 24 h, and changes were observed with or without Ferrostatin-1 (2 μM) treatment, which was applied 2 h before co-culture.

### 2.4 Patients and samples

Peripheral blood samples were prospectively collected from 56 sepsis patients within 24 h of admission and from 32 healthy volunteers. The study adhered to ethical guidelines from the Declaration of Helsinki and received approval from the Ethics Committee of the Second Affiliated Hospital of Harbin Medical University (Approval No.: YJSKY2022-138). Patients included in the study met the Sepsis-3 criteria for sepsis and septic shock. Exclusion criteria included viral myocarditis, severe hepatitis or cirrhosis, incomplete clinical records, malignant tumors, or poor treatment compliance. Healthy volunteers were recruited from routine health examinations through public announcements.

### 2.5 HE staining and lung injury scoring

Paraffin sections were deparaffinized, stained with hematoxylin for 5 min, rinsed, and counterstained with eosin for 10 min, followed by a 15-min rinse. After dehydration and clearing, the sections were mounted in neutral resin for observation and imaging. Two researchers, blinded to the study design, evaluated the HE-stained lung tissue using a previously established scoring method [21].

### 2.6 Tunel staining

Paraffin sections were baked at 60 °C for 2 h, followed by deparaffinization with xylene and alcohol, then rehydrated. After a PBS wash, a hydrophobic barrier was drawn around the sample. Proteinase K solution was applied and incubated at room temperature for 20 min. After washing, 100 μL of Equilibration Buffer was added and incubated for 10–30 min while the labeling solution was prepared. Once equilibrated, the Equilibration Buffer was discarded, and the TdT incubation buffer was applied for 1 h. The sections were washed with PBS (twice for 5 min), counterstained with DAPI, and followed by a final PBS wash (once for 5 min). The sections were then mounted for observation and imaging.

### 2.7 DHE staining

Frozen sections were retrieved from the freezer and brought to room temperature, followed by three washes in PBS. The sections were incubated with a 10 μM dihydroethidium (DHE) fluorescent probe at 37 °C in the dark for 30 min. After nuclear staining, the sections were observed and imaged.

### 2.8 Evans blue staining

Mice were injected with a 0.5 % Evans blue solution via the tail vein at a dose of 2 mL/kg. After 30 min, the mice were sacrificed, and lung tissue was collected. The lungs were placed in 1.5 mL centrifuge tubes, and 1 mL of 50 % trichloroacetic acid (diluted in 1 × PBS) was added. The tissue was homogenized and centrifuged at 10000g for 20 min. The supernatant was diluted fourfold with anhydrous ethanol, and absorbance at 620 nm was measured using a spectrophotometer. The Evans blue concentration was determined using a standard curve.

### 2.9 Adeno-associated virus (AAV) transfection experiment

The macrophage-specific Gbp2 RNA interference adeno-associated virus was obtained from GeneChem Co., Ltd. (Shanghai, China). A dose of 1 × 10^11^ vg/50 μL per mouse was delivered into the lungs via intratracheal instillation. Three weeks later, the mice underwent the planned experimental procedures.

### 2.10 Conditioned medium preparation

Macrophages were treated with LPS (1 μg/mL), with or without pre-treatment with GW4869 (20 μM). The conditioned medium (CM) was then collected and co-cultured with HPMECs to assess cellular changes. THP-1 cells were treated with 50 ng/mL Phorbol 12-myristate 13-acetate (PMA) to induce differentiation into M0 macrophages. LPS was then added to a final concentration of 1 μg/mL. After 24 h, the culture medium was collected and centrifuged at 2000 rpm for 10 min to remove cell debris. The supernatant was filtered through a 0.22 μm filter to obtain the conditioned medium.

### 2.11 EV enrichment and identification

Cell culture supernatant was collected in sterile tubes and centrifuged at 2000g for 20 min at 4 °C to remove cells and debris. The supernatant was centrifuged at 10000g for 30 min at 4 °C to eliminate impurities further. Afterward, the supernatant was subjected to ultracentrifugation at 100000g for 70 min. The resulting pellet, containing the crude EV fraction, was resuspended in pre-cooled PBS and centrifuged again at 100000g for 70 min at 4 °C. The supernatant was discarded, and the EVs were resuspended in pre-cooled PBS to obtain the final EV suspension. Protein concentration was measured using a BCA assay, and EV samples were stored at −80 °C for future use.

EV nanoparticle tracking analysis: EV samples were diluted with 1 × PBS for nanoparticle tracking analysis. After calibration, the samples were diluted with sterile PBS to achieve a particle count between 50 and 400. The dilution factor was entered into the software, and the particle count was verified. The user initiated the analysis by selecting "Measurement" and "Run Video Acquisition," entering the sample details and data storage path, and selecting the appropriate SOP.

Transmission electron microscopy (TEM): EV suspensions were applied to a copper grid and allowed to sit for 1 min, followed by uranyl acetate staining for 1 min. After drying, the samples were imaged using an electron microscope.

### 2.12 EV tracing experiment

In vivo imaging: DiR dye was added to PBS to prepare a working solution, which was then mixed with the EV suspension (100 μL working solution per 1000 μL of EV suspension). The mixture was vortexed and incubated for 30 min. After incubation, PBS was added, and the EVs were enriched by ultracentrifugation to obtain the labeled EVs. Anesthetized mice were administered 50 μL of the labeled EVs via intratracheal instillation. The distribution of EVs in lung tissue was observed using an in vivo imaging system or confocal microscopy.

Immunofluorescence staining (CFSE): According to the manufacturer's instructions and with reference to previous literature [22,23], 5-(and-6)-Carboxyfluorescein Diacetate Succinimidyl Ester (CFSE) staining was used to stain EVs (C34554, Thermo Scientific Fisher). Forty microliters of the CFSE working solution with a concentration of 200 μM was mixed with 15 μL of the PBS suspension containing EVs and then incubated at 37 °C for 2 h. To remove excess dye, the mixture was centrifuged at 10000 g for 70 min, yielding CFSE-labeled EVs.

Immunofluorescence staining (PKH67): A 50 μL EV suspension was mixed with 0.5 mL of Diluent C. Separately, 0.5 mL of Diluent C was mixed with 4 μL of PKH67 dye and then added to the EV suspension. After a 15-min incubation, 1 mL of 0.5 % BSA was added to stop the staining. The EVs were re-purified to obtain PKH67-labeled EVs. These labeled EVs were co-cultured with HPMECs or instilled into the lungs of mice. The distribution of EVs in cells or lung tissue was observed using confocal microscopy.

### 2.13 RNA extraction, reverse transcription, and quantitative PCR

Trizol solution was added to the wells for RNA extraction and incubated at room temperature for 10 min. The cells were resuspended and transferred to RNase-free centrifuge tubes. RNA was extracted by adding chloroform and precipitated with isopropanol. After centrifugation, the supernatant was discarded, and the pellet was washed with ethanol and centrifuged again. The supernatant was removed, and the RNA pellet was air-dried at room temperature before dissolved in RNase-free water. The reverse transcription reaction protocol was as follows: 30 °C for 10 min, 42 °C for 20 min, and 99 °C for 5 min. The resulting product was either used immediately or stored at −20 °C. Detailed primer information is provided in Supplemental Table 1.

### 2.14 Cell transfection

Small interfering RNA (siRNA) was synthesized and purified by GenePharma, with Lipofectamine™ 2000 (purchased from Invitrogen) used as the transfection reagent. Cell confluence was approximately 40–60 % at the time of transfection. The siRNA and transfection reagent were diluted in Opti-MEM, mixed, and briefly incubated. The mixture was then slowly added dropwise to the cell culture medium. After 4–6 h, the medium was replaced with fresh medium, and the cells were subjected to the appropriate treatments. The specific small interfering RNA sequences are provided in Supplemental Table 2.

### 2.15 CCK8 assay

A total of 5000 cells were seeded per well in a 96-well plate for culture. At the time of detection, 10 μL of CCK8 solution was added to each well, and the plate was incubated for 1 h. Absorbance was then measured at 450 nm using a microplate reader.

### 2.16 Flow cytometry analysis

Zombie NIR™ staining was used to evaluate cell death. Endothelial cells were digested with trypsin, washed, and resuspended in 100 μL of Zombie NIR™ working solution (1–10 × 10^6^ cells). The cells were incubated in the dark at room temperature for 30 min, and staining was stopped using 10 % BSA. Emission at 746 nm was detected with a flow cytometer (FC500), and data were analyzed using Kaluza software.

### 2.17 PI staining

The cell culture medium was replaced with staining buffer, and 5 μL of Hoechst and 5 μL of PI reagent were added per milliliter of buffer. Cells were incubated at 4 °C for 30 min. After washing with PBS, images were captured using a fluorescence microscope.

### 2.18 Liperfluo staining

Cells were washed once with serum-free medium after the culture medium was removed. Liperfluo working solution was added, and the cells were incubated for 30 min, followed by two washes. Imaging was performed using a confocal microscope.

### 2.19 JC-1 staining

JC-1 staining was used to assess mitochondrial membrane potential. The JC-1 solution was diluted 1:200 in the staining buffer to prepare the working solution. Cells were incubated with the JC-1 working solution at 37 °C for 20 min, then washed with staining buffer. Observations and imaging were performed using a confocal microscope.

### 2.20 ROS staining

DCFH-DA was diluted in DMEM to a final concentration of 10 μM. The cell culture medium was replaced with the DCFH-DA working solution and incubated in the dark at 37 °C for 30 min. After removing the probe, Hoechst was used for nuclear staining, and ROS levels were observed and imaged using a fluorescence microscope.

### 2.21 Flow cytometry with the C11 BODIPY probe

Detection of the intracellular lipid peroxidation level was carried out using the C11-BODIPY probe (D3861, Thermo Scientific Fisher). A 10 mM stock solution of C11-BODIPY 581/591 was prepared using DMSO (D2650, Sigma-Aldrich). C11-BODIPY 581/591 with a final concentration of 10 μM was added to the cell culture medium. After incubation at 37 °C in the dark for 30 min, the cells were washed three times with PBS to remove the excess dye. Subsequently, the cells were digested with 0.25 % trypsin and collected. After being washed twice again with PBS, the cells were resuspended in PBS. Immediately, the mean fluorescence intensity (MFI) of cells from each group at 488 nm was detected with a nanoflow cytometer (Micro Plus, Apogee).

### 2.22 Western blot analysis

Protein samples were loaded and separated by SDS-PAGE. The gel was cut and transferred to a 0.22 μm PVDF membrane at 300 mA for 2 h under ice bath conditions. The membrane was blocked with 5 % non-fat milk for 1 h, then incubated with the primary antibodies: ZO-1 (1:2000, AF5145, Affinity, OH, USA), VE-Cadherin (1:2000, AF6265, Affinity, OA, USA), occludin (1:1000, 91131, Cell Signaling Technology, MA, USA), β-actin (1:100000, AC026, ABclonal, Wuhan, China), GAPDH (1:2500, ab9485, Abcam, Cambridge, UK), SLC7A11 (1:10000, ab175186, Abcam, Cambridge, UK), GPX4 (1:10000, ab125066, Abcam, Cambridge, UK), Calnexin (1:2000, AF5362, Affinity, OA, USA), TSG101 (1:3000, DF8427, Affinity, OA, USA), CD81 (1:2000, DF2306, Affinity, OA, USA), CD63 (1:1000, AF5117, Affinity, OA, USA), GBP2 (1:2000, Proteintech, 11854-1-AP, IL, USA), APO B48 (1:10000, ab139401, Abcam, Cambridge, UK) and Ub (1:2000, PTM-1107, Jingjie, Hangzhou, China). After washing with TBST, the membrane was incubated with the secondary antibodies (1:10000, ab6712, Abcam, Cambridge, UK; 1:100000, AS003, ABclonal, Wuhan, China) for 1 h, rewashed, and developed using ECL for imaging.

### 2.23 Cellular thermal shift assay (CETSA)

The interaction between PD and GBP2 was confirmed using CETSA. Cell lysates treated with either vehicle or PD were divided into ten equal portions and heated at 37 °C–64 °C. The supernatant was collected for further analysis after two cycles of freezing and thawing with liquid nitrogen [24].

### 2.24 Virtual screening

The L6000 natural product library (L6000-Targetmol-Natural Product Library for HTS–4320 cpds) from Topscience was used for virtual screening. The 2D sdf files were imported into Schrödinger software, where the LigPrep module was utilized to prepare 3D structures for each compound using the OPLS_4 force field. The Epik module was used to determine all possible stereoisomers and protonation states.

The docking grid center was set to the crystallized ligand at the small molecule GDP binding pocket. The outer box size was adjusted to match the crystallized ligand, and the inner box size was set to 10 Å. Molecular docking was performed using Schrödinger's Virtual Screening Workflow module, with the LigPrep-prepared 3D structures serving as the screening database.

The virtual screening process followed four steps: (1) HTVS (High-Throughput Virtual Screening): All stereoisomeric states of each ligand were retained, and one conformer per stereoisomer was generated. The top 50 % of compounds based on score were selected for further screening; (2) SP (Standard Precision): The best-scoring stereoisomer of each ligand was retained, with one conformer generated per stereoisomer. The top 20 % of compounds were selected for the next round; (3) XP (Extra Precision): Only the best-scoring stereoisomer of each ligand was retained, and one conformer was generated. The top 100 compounds were selected for further analysis; (4) MM-GBSA: Final compounds were rescored using binding free energy calculations. After removing duplicates and compounds with MM-GBSA scores above −50 kcal/mol.

### 2.25 Molecular dynamics simulation

Molecular dynamics simulations were conducted using Gromacs 2022.3. PD was pre-processed with AmberTools22, applying the GAFF force field, and hydrogen atoms were added using Gaussian 16W to calculate RESP charges, which were then incorporated into the system's topology file. The simulations were performed at a constant temperature of 300K and pressure of 1 bar, using the Amber99sb-ildn force field. Water molecules (Tip3p model) were used as the solvent, and Na^+^ ions were added to neutralize the system's charge [25,26].

The system first underwent energy minimization via the steepest descent method, followed by 100,000 steps each of NVT (constant volume) and NPT (constant pressure) equilibration, with a coupling constant of 0.1 ps and a duration of 100 ps. A free molecular dynamics simulation was then carried out for 5,000,000 steps with a two fs time step, totaling 100 ns. After the simulation, Gromacs tools were used to analyze the trajectory, calculating the root mean square deviation (RMSD) and root mean square fluctuation (RMSF) of amino acid motion.

### 2.26 Detection of GTPase activity

The activity of GTPase of GBP2 was determined using a modified malachite green reagent (ab272520, Abcam). Neither the enzyme preparation nor the assay buffer contained free phosphate. Phosphate standards with concentrations ranging from 0 to 50 μM were prepared to construct a standard curve. GBP2 at concentrations of 0, 20, 40, 80, 100, and 120 nM, PD at concentrations of 0, 0.625, 1.25, 2.5, 5, 10, and 20, as well as a combination of 100 nM GBP2 and PD with increasing concentrations were used respectively. The absorbance was measured at a wavelength of 620 nm, and the content of released phosphate was calculated to evaluate the GTPase activity.

### 2.27 Statistical analysis

Data were analyzed and graphs were generated using GraphPad Prism 9.0 and Image J software. Results are expressed as the mean ± standard deviation from at least three independent experiments. Differences between the two groups were evaluated using an independent samples *t*-test, while multiple group comparisons were performed using one-way ANOVA with Tukey's post hoc test. A *P*-value of <0.05 was considered statistically significant, with ∗ indicating *P* < 0.05, ∗∗*P* < 0.01, ∗∗∗*P* < 0.001, and ∗∗∗∗*P* < 0.0001.

## 3 Results

### 3.1 Conditioned medium from LPS-stimulated macrophages induces ferroptosis in pulmonary microvascular endothelial cells

**Fig. 1:**
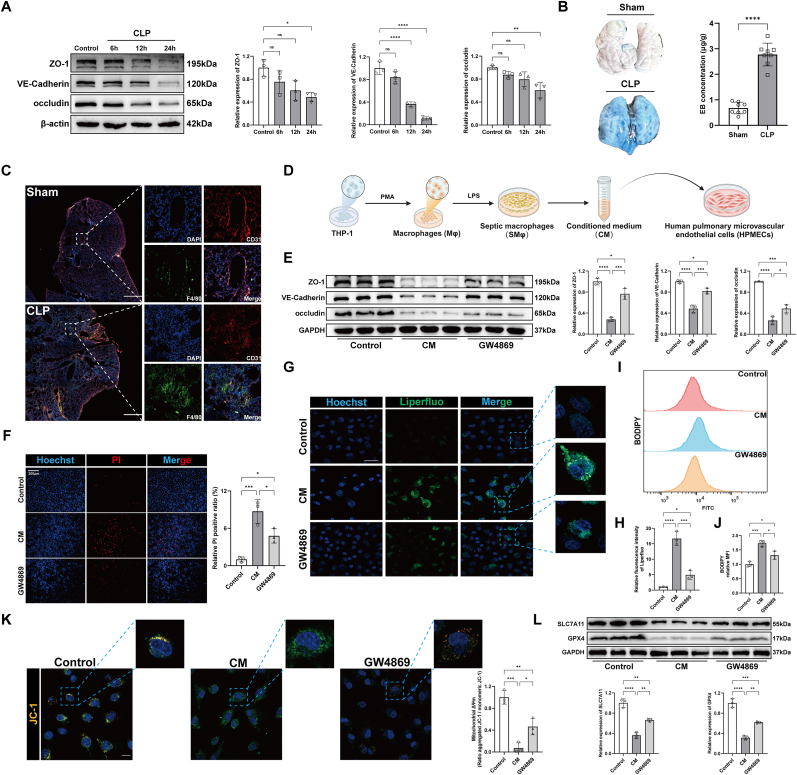
Conditioned medium from LPS-stimulated macrophages induces ferroptosis in pulmonary microvascular endothelial cells. (A) Representative immunoblotting and quantification of barrier-associated proteins (ZO-1, VE-Cadherin, and occludin) in the lungs of mice either untreated or subjected to CLP surgery for 6h, 12h, and 24h (n = 3). (B) Representative images and quantification of Evans blue staining in mouse lungs (n = 8). (C) Representative immunofluorescence images of DAPI/CD31/F4/80 staining in mouse lungs. Scale bar = 500 μm. (D) Schematic diagram illustrating the co-culture of septic macrophage-conditioned medium (CM) with human pulmonary microvascular endothelial cells (HPMECs). (E) Representative immunoblotting and quantification of barrier-associated proteins in HPMECs co-cultured with CM, with or without GW4869 treatment (n = 3). (F) Representative images and quantification of PI staining in HPMECs (n = 3). Scale bar = 200 μm. (G, H) Representative images and quantification of liperfluo staining in HPMECs (n = 3). Scale bar = 50 μm. (I, J) C11-BODIPY assay evaluating the lipid ROS levels in HPMECs (n = 3). (K) Representative images for mitochondrial membrane potential of HPMECs (n = 3). Scale bar = 50 μm. (L) Representative immunoblotting and quantification of SLC7A11 and GPX4 in HPMECs (n = 3). Data are presented as mean ± SD. ∗*P* < 0.05, ∗∗*P* < 0.01, ∗∗∗*P* < 0.001, and ∗∗∗∗*P* < 0.0001.

To evaluate changes in lung vascular permeability in CLP mice, lung tissue samples were collected 6, 12, and 24 h post-surgery. Western blot analysis revealed a significant reduction in vascular barrier-related proteins in the lungs of CLP mice at 24 h compared to controls (Fig. 1A), indicating pulmonary endothelial barrier damage. Evans blue staining confirmed increased lung vascular permeability in the CLP group (Fig. 1B). Immunofluorescence staining showed a significant increase in F4/80^+^ cells in the lung tissue of CLP mice compared to the sham group, with these cells closely positioned to CD31^+^ cells (Fig. 1C). THP-1 cells were differentiated into macrophages (Mφ) using PMA, followed by LPS treatment to mimic the septic microenvironment and generate sepsis-associated macrophages (SMφ). After LPS treatment, macrophages transitioned from a round to a spindle shape, forming long projections (Supplemental Fig. 1A). Compared to PBS-treated cells, LPS-treated cells showed significantly higher mRNA levels of the pro-inflammatory cytokines IL-6, IL-1β, and TNF-α, indicating an enhanced inflammatory response (Supplemental Figs. 1B–D). As shown in Fig. 1D, conditioned medium (CM) from SMφ was collected after 24 h and co-cultured with HPMECs. The CM-treated HPMECs showed decreased expression of vascular barrier-related proteins compared to controls (Fig. 1E). Additionally, CM treatment resulted in a higher percentage of PI-positive cells (Fig. 1F) and elevated lipid peroxidation levels (Fig. 1G–J) in HPMECs, along with a significant reduction in mitochondrial membrane potential (Fig. 1K). Additionally, ferroptosis markers SLC7A11 and GPX4 were significantly downregulated in the CM group compared to controls (Fig. 1L). Pre-treatment of SMφ with the EV synthesis/secretion inhibitor GW4869 significantly reversed these changes in HPMECs. These findings suggest that CM induces ferroptosis in HPMECs, with EVs serving as key mediators.

### 3.2 SMφ-derived EVs induce ferroptosis in pulmonary microvascular endothelial cells

**Fig. 2:**
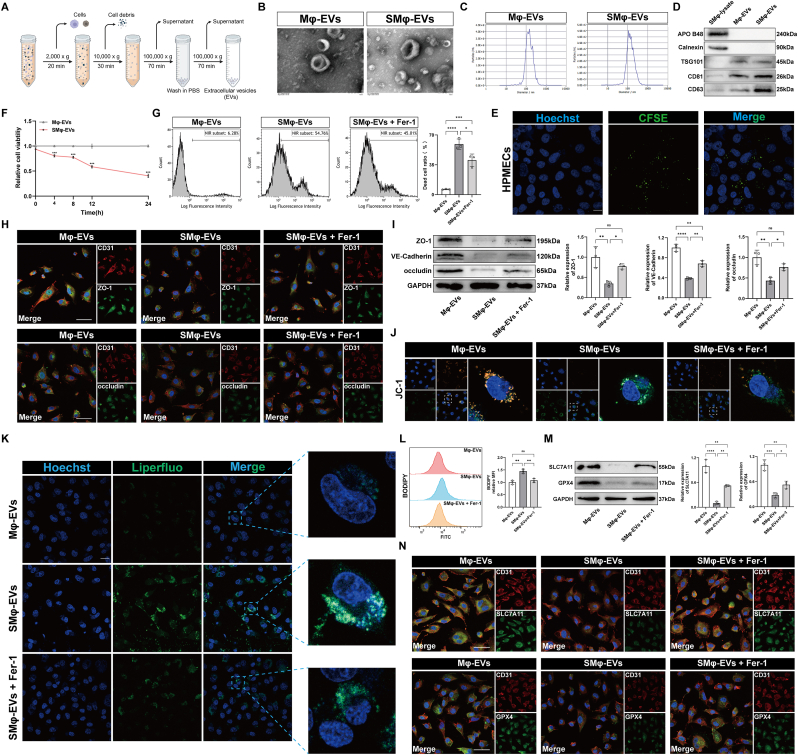
SMφ-derived EVs induce ferroptosis in pulmonary microvascular endothelial cells. (A) The flowchart of EV enrichment by ultracentrifugation. (B) Transmission electron microscopy (TEM) images of EVs isolated from the medium of Mφ and SMφ. Scale bar = 100 nm. (C) Representative nanoparticle tracking analysis images showing the distribution and average diameter of EVs. (D) Representative immunoblotting of EV markers. (E) Representative immunofluorescence staining of Hoechst/CFSE after co-culture with human pulmonary microvascular endothelial cells (HPMECs) using CFSE-labeled EVs. Scale bar = 10 μm. (F) Quantification of CCK8 assays performed on HPMECs co-cultured with Mφ-EVs and SMφ-EVs (n = 5). (G) Representative images and quantification of the Zombie NIR™ staining in HPMECs co-cultured with Mφ-EVs and SMφ-EVs in the presence or absence of ferrostatin-1 (Fer-1) (n = 3). (H) Representative immunofluorescence staining for DAPI/CD31/ZO-1 and DAPI/CD31/occludin in HPMECs. Scale bar = 50 μm. (I) Representative immunoblotting and quantification of barrier-associated proteins (ZO-1, VE-Cadherin and occludin) in HPMECs (n = 3). (J) Representative images of mitochondrial membrane potential of HPMECs. Scale bar = 50 μm. (K) Representative images of liperfluo staining in HPMECs. Scale bar = 50 μm. (L) C11-BODIPY assay evaluating the lipid ROS levels in HPMECs (n = 3). (M) Representative immunoblotting and quantification of SLC7A11 and GPX4 in HPMECs (n = 3). (N) Representative images of immunofluorescence staining for DAPI/CD31/SLC7A11 and DAPI/CD31/GPX4 in HPMECs. Scale bar = 50 μm. Data are presented as mean ± SD. ∗*P* < 0.05, ∗∗*P* < 0.01, ∗∗∗*P* < 0.001, and ∗∗∗∗*P* < 0.0001.

EVs were isolated from the culture media of Mφ and SMφ cells (Mφ-EVs and SMφ-EVs) via ultracentrifugation (Fig. 2A). Both Mφ-EVs and SMφ-EVs exhibited a characteristic biconcave, double-layer membrane structure (Fig. 2B). Nanoparticle tracking analysis (NTA) showed that Mφ-EVs and SMφ-EVs had similar concentrations and particle sizes, with an approximate diameter of 140 nm (Fig. 2C). They expressed the marker proteins TSG101, CD81, and CD63, but lacked Calnexin and APO B48 (Fig. 2D), confirming their EV origin. When CFSE- or PKH67-labeled EVs were co-cultured with HPMECs, the EVs were absorbed by the cells (Fig. 2E and Supplemental Fig. 2A). Co-culture of Mφ-EVs and SMφ-EVs with HPMECs, followed by viability assessment using CCK8 assay, revealed that SMφ-EVs progressively reduced HPMEC viability over time. Significant differences in cell viability between the SMφ-EVs and Mφ-EVs groups were observed at 4, 8, 12, and 24 h (Fig. 2F). Flow cytometry analysis indicated a significantly higher ratio of cell death in the SMφ-EVs group compared to the Mφ-EVs group. However, pre-treatment of HPMECs with the ferroptosis inhibitor Ferrostatin-1 (Fer-1) reduced the cell death ratio (Fig. 2G). Additionally, SMφ-EVs-treated HPMECs showed lower expression of barrier-related proteins (Fig. 2H and I) and mitochondrial membrane potential (Fig. 2J and Supplemental Fig. 2B) and increased lipid peroxidation levels (Fig. 2K and L, and Supplemental Fig. 2C). The ferroptosis-related proteins SLC7A11 and GPX4 expression was also downregulated (Fig. 2M and N). These effects were partially reversed by Fer-1. In summary, septic macrophage-derived EVs were found to induce ferroptosis and disrupt the barrier integrity of pulmonary microvascular endothelial cells.

### 3.3 EVs derived from LPS-treated RAW264.7 cells mediate ferroptosis in mouse lungs

**Fig. 3:**
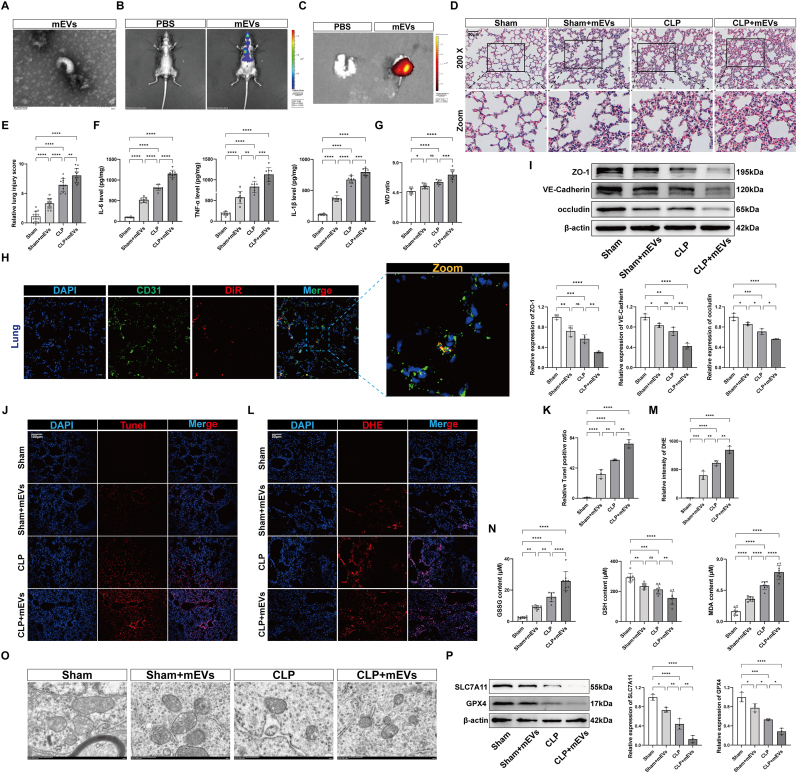
EVs derived from LPS-treated RAW264.7 cells mediate ferroptosis in mouse lungs. (A) Transmission electron microscopy (TEM) images of the isolated EVs from the LPS-treated RAW264.7 cells. Scale bar = 100 nm. (B–C) In vivo fluorescence imaging (B) and in ex vivo fluorescence images (C) of lung tissue in mice with or without DiR-labeled EVs. (D) Representative HE-stained images of sham or CLP mice with or without mEVs. Scale bar = 50 μm. (E) Quantification of lung injury scores in mice. (F) Quantification of IL-6, TNF-α, and IL-1β levels in mouse lungs (n = 8). (G) Wet to dry weight ratio of mouse lungs (n = 8). (H) Representative immunofluorescence staining for DAPI/CD31/DiR in mouse lungs after intratracheal injection of DiR-labeled EVs. Scale bar = 20 μm. (I) Representative immunoblotting and quantification of barrier-associated proteins (ZO-1, VE-Cadherin, and occludin) in mouse lungs (n = 3). (J, K) Representative images of Tunel staining of mouse lungs and quantification of Tunel-positive cells (n = 3). Scale bar = 100 μm. (L, M) Representative images of DHE staining of mouse lungs and quantification of DHE fluorescence intensity (n = 3). Scale bar = 50 μm. (N) Quantification of GSSG, GSH, and MDA levels in mouse lungs (n = 8). (O) TEM image of mouse lungs. Scale bar = 500 nm. (P) Representative immunoblotting and quantification of SLC7A11 and GPX4 in mouse lungs (n = 3). Data are presented as mean ± SD. ∗*P* < 0.05, ∗∗*P* < 0.01, ∗∗∗*P* < 0.001, and ∗∗∗∗*P* < 0.0001.

To investigate the in vivo effects of macrophage-derived EVs, RAW264.7 cells were treated with LPS, and the EVs (mEVs) were isolated (Fig. 3A and Supplemental Figs. 3A and B). DiR-labeled EVs were intratracheally instilled into mice, and both in vivo and ex vivo imaging demonstrated mEV distribution in the lungs (Fig. 3B and C). Lung tissue samples were collected 24 h post-instillation for further analysis. Compared to the sham group, the sham + mEVs group displayed thickened lung interstitium, increased inflammatory cell infiltration and injury scores, elevated inflammatory markers, and a higher lung wet-to-dry ratio. The CLP + mEVs group exhibited more severe lung injury, inflammation, and edema compared to the CLP group (Fig. 3D–G). The co-localization of DiR- or PKH67-labeled mEVs with CD31^+^ cells was observed following endotracheal instillation, indicating mEV uptake by lung vascular endothelial cells (Fig. 3H and Supplemental Fig. 3C). Western blot analysis revealed significant downregulation of barrier-related proteins in lungs from the sham + mEVs, CLP, and CLP + mEVs groups, with further reductions in the CLP + mEVs group compared to the CLP group (Fig. 3I). Increased ratio of Tunel-positive cells, elevated superoxide anion fluorescence, and higher levels of oxidative stress markers (GSSG, GSH, and MDA) were found in the sham + mEVs, CLP, and CLP + mEVs groups compared to the sham group, with more pronounced changes in the CLP + mEVs group (Fig. 3J–N). TEM revealed shrinkage and increased membrane density of mitochondria in the lungs of these groups (Fig. 3O). Additionally, SLC7A11 and GPX4 protein levels were significantly reduced in the sham + mEVs, CLP, and CLP + mEVs groups compared to the sham group, with a further reduction in the CLP + mEVs group compared to the CLP group (Fig. 3P). These results indicate that macrophage-derived EVs induce ferroptosis in mouse lungs during sepsis, exacerbating lung injury.

### 3.4 GBP2 in SMφ-derived EVs is a potential mediator of pulmonary endothelial cell injury

**Fig. 4:**
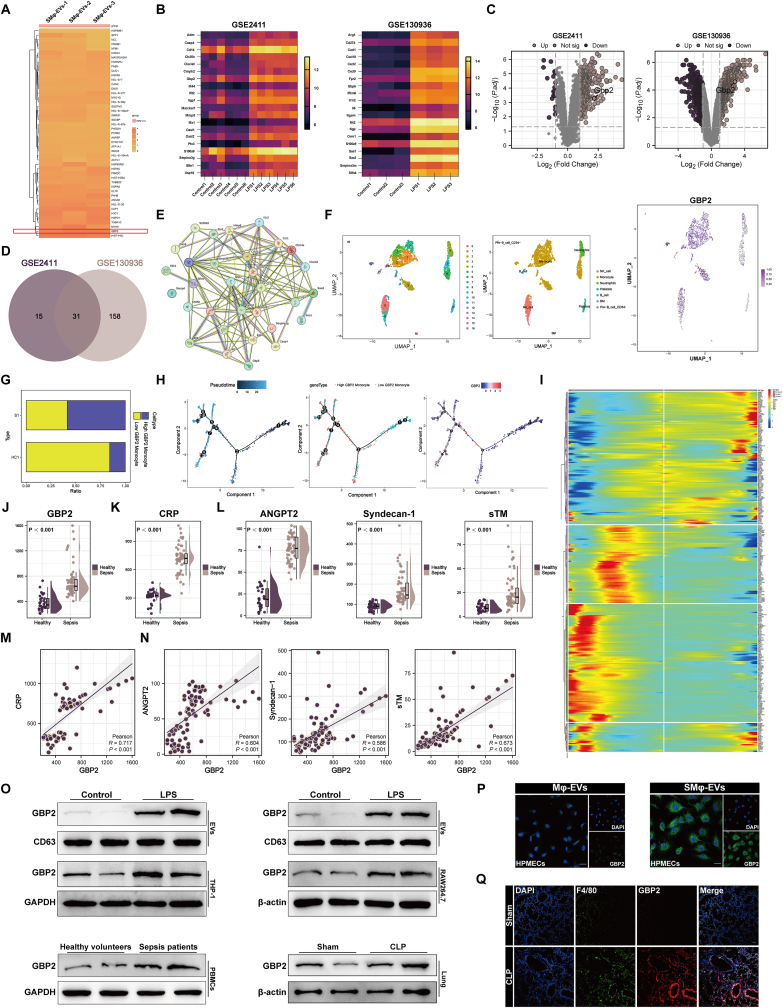
GBP2 in SMφ-derived EVs is a potential mediator of pulmonary endothelial cell injury. (A) Heatmap of SMφ-EVs proteomics sequencing. (B–C) Heatmaps (B) and volcano plots (C) and of genes in the GSE2411 and GSE130936 datasets. (D) Venn diagram of differentially expressed genes (DEGs) for the GSE2411 and GSE130936 datasets. (E) Protein-protein interaction network diagram. (F) UMAP plot of cells clustered according to the expression of known marker genes and scatter plot showing GBP2 expression in seven cell types. (G) Proportional graph displaying the proportion of high and low GBP2 expression in monocytes of sepsis patients and healthy volunteers. (H) Cell trajectories displayed according to pseudotime, which are chronologically ordered based on dark-to-light color indications, and expression distribution of GBP2 in the pseudotime analysis. (I) Heatmap for BEAM analysis of monocytes. (J–L) Levels of GBP2 (J) in EVs from plasma of healthy volunteers (n = 32) and sepsis patients (n = 56), as well as levels of CRP (K) and markers of vascular injury (ANGPT2, Syndecan-1 and sTM) (L) in plasma. (M–N) Correlation of GBP2 levels in EVs from plasma with levels of CRP (M) and markers of vascular injury (ANGPT2, Syndecan-1 and sTM) (N) in plasma. (O) Representative immunoblotting of GBP2 in THP-1 and RAW264.7 cells and EVs secreted by both, in EVs from clinical samples, and in mouse lungs. (P) Representative images of immunofluorescence staining for DAPI/GBP2 in HPMECs treated with Mφ-EVs and SMφ-EVs. Scale bar = 20 μm. (Q) Representative images of immunofluorescence staining for DAPI/F4/80/GBP2 in lung tissue of mice with or without CLP operation. Scale bar = 50 μm. Data are presented as mean ± SD. ∗*P* < 0.05, ∗∗*P* < 0.01, ∗∗∗*P* < 0.001, and ∗∗∗∗*P* < 0.0001.

To identify key molecules involved in macrophage-derived EV-mediated endothelial cell injury, proteomics sequencing was performed to assess the protein components of SMφ-EVs (Fig. 4A). Simultaneously, differential expression analysis was performed on si-ALI-related datasets GSE2411 and GSE130936 from the GEO database. The intersection of the two datasets identified 31 differentially expressed genes (DEGs) associated with si-ALI (Fig. 4B–D). Protein-protein interaction network analysis of these DEGs was performed, and the key molecules identified were compared with the proteomics results (Fig. 4E). Based on these findings and previous research, GBP2 emerged as a potential molecule in SMφ-derived EVs accounting for the progression of si-ALI. Reanalysis of previously published scRNA-Seq data from sepsis patients (GSE167363) revealed that a higher proportion of monocytes expressed elevated levels of GBP2 in sepsis patients (Fig. 4F and G). Pseudotime analysis showed that monocytes with low GBP2 expression were located at the initial position (Fig. 4H and I). Additionally, GBP2 was significantly enriched in the plasma EVs of sepsis patients (Fig. 4J), and its expression positively correlated with CRP levels and vascular injury markers, including ANGPT2, Syndecan-1, and sTM in peripheral blood (Fig. 4K–N), suggesting a potential link between GBP2 expression and vascular barrier disruption. To further investigate the clinical significance of GBP2, this study analyzed the demographic and clinical characteristics of sepsis patients, stratified into high and low GBP2 expression groups based on the median GBP2 expression level (Supplemental Table 3). The results showed that the two groups had similar baseline characteristics and laboratory parameters. However, patients with high GBP2 expression exhibited significantly elevated D-dimer levels and higher SOFA scores. These findings suggest that GBP2 upregulation may be positively correlated with disease severity in sepsis patients.

GBP2 levels were also elevated in LPS-treated THP-1 and RAW264.7 cells and their secreted EVs (Fig. 4O and Supplemental Figs. 4A–D). Similarly, GBP2 was highly expressed in the peripheral blood monocytes of sepsis patients and the lung tissue of CLP- or LPS-treated mice (Fig. 4O and Supplemental Fig. 4F). Furthermore, GBP2 levels were higher in HPMECs co-cultured with SMφ-EVs than in those co-cultured with Mφ-EVs, with predominant localization in the cytoplasm, indicating that GBP2 can be transferred into endothelial cells via EVs (Fig. 4P). GBP2 was also highly expressed in macrophages in the lungs of CLP- and LPS-treated mice (Fig. 4Q and Supplemental Fig. 4E). Immunofluorescence analysis further revealed a time-dependent increase in GBP2 expression and a progressive decrease in GPX4 in pulmonary vascular endothelial cells of CLP mice (Supplemental Figs. 4G and H). In conclusion, these findings suggest that GBP2 is a potential mediator in macrophage-derived EV-mediated vascular endothelial injury during si-ALI.

### 3.5 Macrophage-derived EVs promote pulmonary vascular endothelial ferroptosis and disruption via GBP2 in si-ALI

**Fig. 5:**
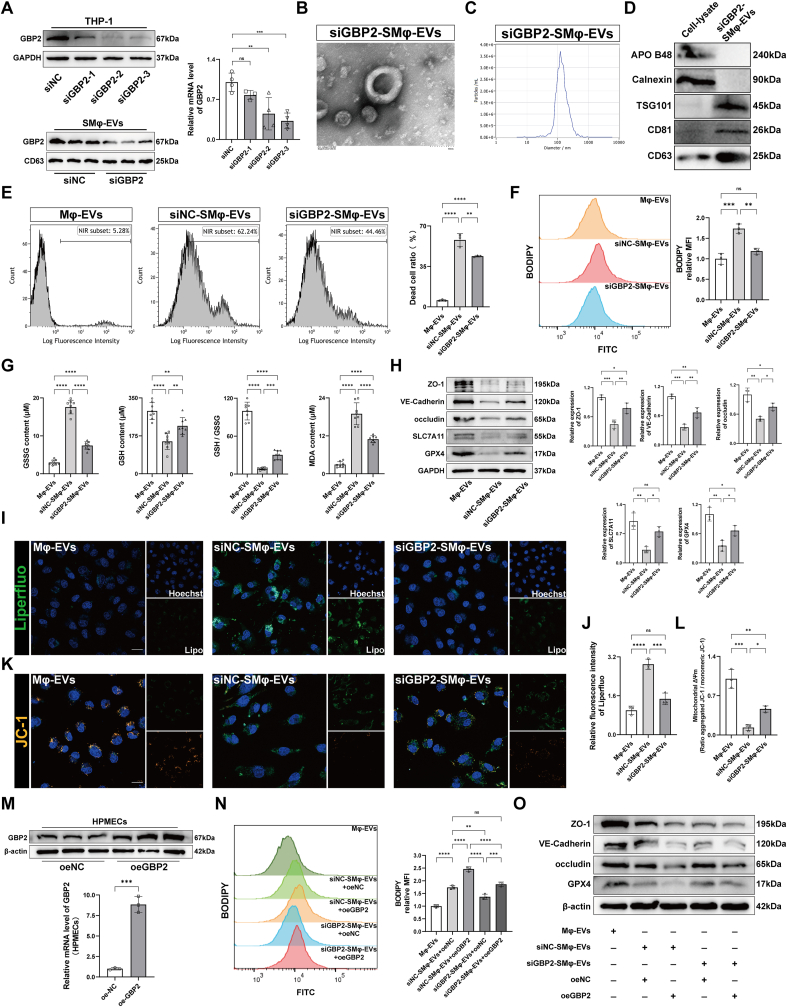
Macrophage-derived EVs promote pulmonary vascular endothelial ferroptosis and disruption via GBP2 in si-ALI. (A) Representative immunoblotting for efficiency validation after silencing GBP2 in THP-1 cells and SMφ-EVs and RT-qPCR analysis to determine mRNA expression of GBP2 in THP-1 cells (n = 4). (B) Transmission electron microscopy images of the isolated EVs of SMφ after silencing GBP2. Scale bar = 100 nm. (C) Representative nanoparticle tracking analysis images showing the distribution and average diameter of mEVs. (D) Representative immunoblotting of mEV markers. (E) Representative images and quantification of the Zombie NIR™ staining in HPMECs co-cultured with Mφ-EVs and SMφ-EVs with or without silencing GBP2 (n = 3). (F) C11-BODIPY assay evaluating the lipid ROS levels in HPMECs (n = 3). (G) Quantification of GSSG, GSH, GSH/GSSG and MDA in HPMECs (n = 8). (H) Representative immunoblotting and quantification of barrier-associated proteins (ZO-1, VE-Cadherin and occludin) and ferroptosis-associated proteins (SLC7A11 and GPX4) in HPMECs (n = 3). (I, J) Representative images of liperfluo staining (I) and quantitative analysis (J) of liperfluo fluorescence intensity in HPMECs (n = 3). Scale bar = 50 μm. (K, L) Representative images (K) and quantification (L) for mitochondrial membrane potential of HPMECs (n = 3). (M) Representative immunoblotting for efficiency validation after overexpressing GBP2 in HPMECs and RT-qPCR analysis to determine mRNA expression of GBP2 in HPMECs (n = 3). (N) C11-BODIPY assay evaluating the lipid ROS levels in HPMECs (n = 3). (O) Representative immunoblotting of barrier-associated proteins (ZO-1, VE-Cadherin and occludin) and ferroptosis-associated proteins GPX4 in HPMECs (n = 3). Data are presented as mean ± SD. ∗*P* < 0.05, ∗∗*P* < 0.01, ∗∗∗*P* < 0.001, and ∗∗∗∗*P* < 0.0001.

In the in vitro experiments, three RNA interference sequences were designed to silence GBP2 expression in THP-1 cells, and the effective sequence was selected (Fig. 5A). EVs were isolated from SMφ transfected with siGBP2 (siGBP2-SMφ-EVs) and co-cultured with HPMECs (Fig. 5B–D). The siNC-SMφ-EVs group exhibited significantly higher cell death, oxidative stress, and lipid ROS levels compared to the Mφ-EVs group (Fig. 5E–G). However, these indicators were reduced in the siGBP2-SMφ-EVs group relative to the siNC-SMφ-EVs group (Fig. 5E–G). In the siNC-SMφ-EVs group, the expression of barrier and ferroptosis-related proteins and mitochondrial membrane potential was significantly lower than in the Mφ-EVs group, while lipid peroxidation and ROS levels were elevated. These changes were partially reversed by silencing GBP2 (Fig. 5H–L and Supplemental Fig. 5A). Interestingly, this study also found that apoptotic marker expression was significantly higher in the siNC-SMφ group than in the Mφ-EVs group. However, this increase was markedly reversed following GBP2 silencing in macrophages (Supplemental Fig. 5B). Furthermore, GBP2 was overexpressed in HPMECs, and the overexpression efficiency was confirmed by Western blot and RT-qPCR (Fig. 5M). The cells were then co-cultured with siGBP2-SMφ-EVs or siNC-SMφ-EVs. The results showed that GBP2 overexpression in HPMECs partially restored the ability of siGBP2-SMφ-EVs to increase lipid ROS levels and reduce the expression of barrier and ferroptosis-related proteins (ZO-1, VE-Cadherin, occludin, and GPX4) in HPMECs (Fig. 5N and O, and Supplemental Fig. 5C).

Subsequently, GBP2 was overexpressed in THP-1 cells, and the overexpression efficiency was confirmed (Supplemental Fig. 5D). EVs from SMφ overexpressing GBP2 (oeGBP2-SMφ-EVs) were isolated and co-cultured with HPMECs. Compared to the Mφ-EVs group, the oeNC-SMφ-EVs group exhibited a significant rise in lipid ROS and decreased expression of barrier and ferroptosis-related proteins. These effects were even more pronounced in the oeGBP2-SMφ-EVs group compared to the oeNC-SMφ-EVs group (Supplemental Figs. 5E–H).

In the in vivo experiments, EVs from LPS-treated RAW264.7 cells transfected with either siGbp2 or oeGbp2 (siGbp2-mEVs or oeGbp2-mEVs) were administered to mice via intratracheal instillation (Supplemental Figs. 6A–G). HE staining and lung injury scoring indicated that lung damage was significantly reduced in the CLP + siGbp2-mEVs group compared to the CLP + siNC-mEVs group (Supplemental Fig. 6H). In contrast, the CLP + oeGbp2-mEVs group showed significantly greater lung injury compared to the CLP + oeNC-mEVs group (Supplemental Fig. 6I). Tunel and DHE staining demonstrated that the increased cell death and superoxide anion levels in the lungs of CLP mice induced by mEVs were mitigated after GBP2 silencing, whereas both were further elevated following GBP2 overexpression (Supplemental Figs. 6J and K). Furthermore, the expression of barrier and ferroptosis-related proteins in lung tissue was significantly upregulated in the CLP + siGbp2-mEVs group compared to the CLP + siNC-mEVs group (Supplemental Fig. 6L). Conversely, these protein levels were significantly downregulated in the CLP + oeGbp2-mEVs group compared to the CLP + oeNC-mEVs group (Supplemental Fig. 6L). These results suggest that GBP2 is crucial in macrophage-derived EV-promoted ferroptosis in pulmonary vascular endothelial cells and lung injury during sepsis.

### 3.6 Knockdown of Gbp2 in macrophage alleviates lung injury of CLP mice

**Fig. 6:**
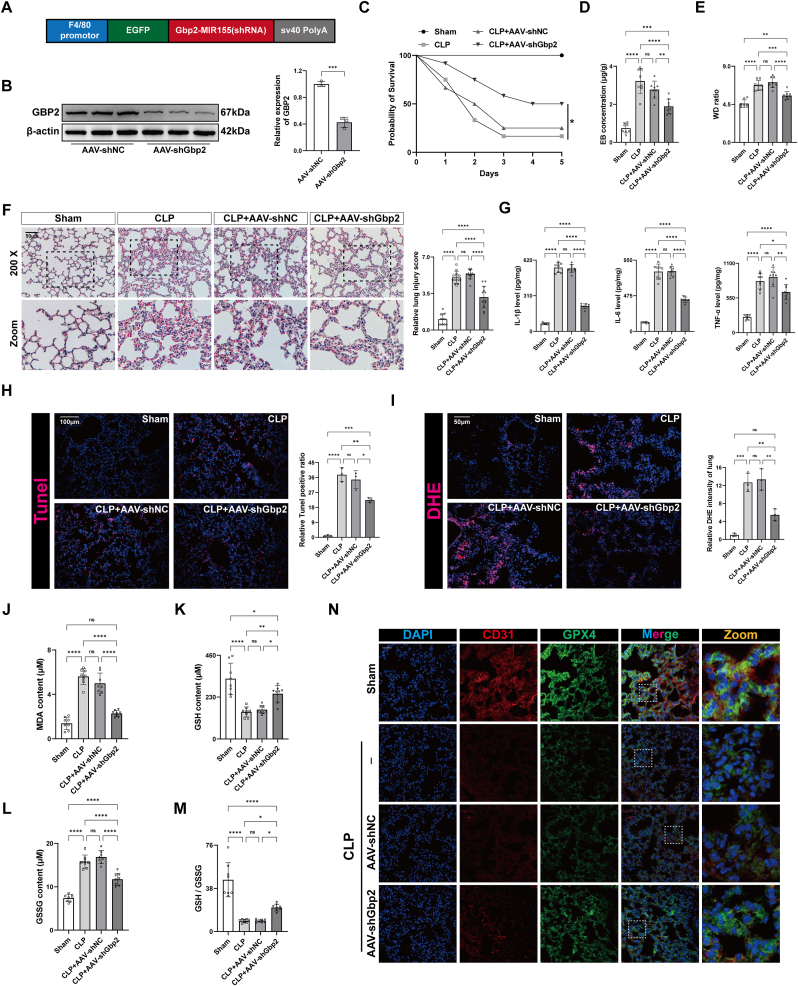
Knockdown of Gbp2 in macrophage alleviates lung injury of CLP mice. (A) Schematic representation of AAV vector construction targeting the knockdown of GBP2 in macrophages. (B) Representative images and quantification of immunoblotting of GBP2 in mouse lungs after intratracheal injection of AAV-shNC and AAV-shGbp2 (n = 3). (C) Survival rate of mice treated with or without CLP after receiving or not receiving intratracheal injection of AAV-shNC or AAV-shGbp2 (n = 12). (D) Relative content of Evans blue in mouse lungs (n = 8). (E) Wet to dry weight ratio of mouse lungs (n = 6). (F) Representative images of HE staining of mouse lungs and lung injury score. Scale bar = 50 μm. (G) Quantification of IL-6, TNF-α and IL-1β in mouse lungs (n = 8). (H, I) Representative images and quantification of Tunel (H) and DHE (I) staining of mouse lungs (n = 3). Scale bar = 100 μm or 50 μm. (J–M) Quantification of MDA (J), GSH (K), GSSG (L) and GSH/GSSG (M) in mouse lungs (n = 8). (N) Representative images of immunofluorescence staining for DAPI/CD31/GPX4 in mouse lungs. Scale bar = 20 μm. Data are presented as mean ± SD. ∗*P* < 0.05, ∗∗*P* < 0.01, ∗∗∗*P* < 0.001, and ∗∗∗∗*P* < 0.0001.

To evaluate the in vivo effects of Gbp2 knockdown, a macrophage-specific AAV vector was used for intratracheal administration of shGbp2 (Fig. 6A). This approach significantly reduced Gbp2 protein levels in the lung tissue of mice (Fig. 6B and Supplemental Fig. 7A), with no effect on normal lungs (Supplemental Fig. 7B) and increased survival rates within five days post-CLP surgery (Fig. 6C). Lung permeability analysis revealed that Gbp2 knockdown in macrophages significantly reduced Evans blue content in the lungs of CLP mice and alleviated pulmonary edema, indicating enhanced vascular barrier integrity (Fig. 6D and E). AAV-shGbp2 treatment also significantly alleviated lung injury in CLP mice (Fig. 6F). Furthermore, AAV-shGbp2 treatment significantly mitigated the elevated levels of inflammatory cytokines, cell death ratio, and redox imbalance induced by CLP (Fig. 6G–M). Immunofluorescence analysis confirmed that the proportion of CD31^+^/GPX4^+^ cells in the lungs of the CLP + AAV-shGbp2 group was significantly higher than in the CLP or CLP + AAV-shNC groups (Fig. 6N). These results suggest that macrophage-specific Gbp2 knockdown effectively alleviates lung injury and ferroptosis.

### 3.7 Macrophagic GBP2 exacerbates lung injury by disrupting the endothelial barrier via downregulating GPX4 in endothelial cells

**Fig. 7:**
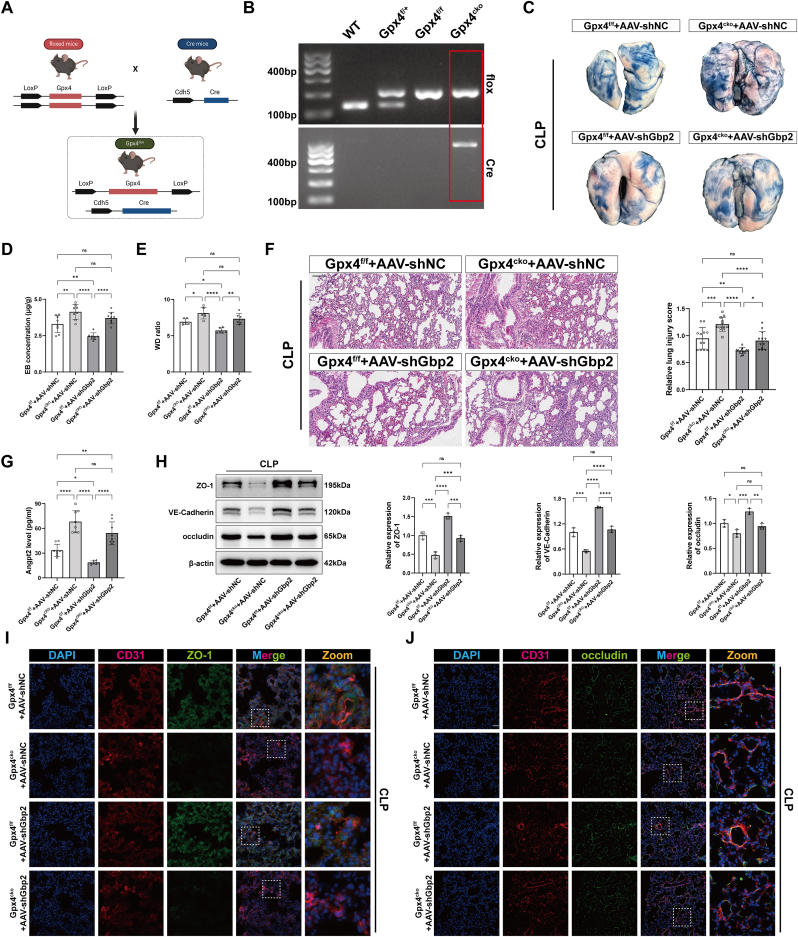
Macrophagic GBP2 exacerbates lung injury by disrupting the endothelial barrier via downregulating GPX4 in endothelial cells. (A) Schematic diagram of the construction of Gpx4^cko^ mouse. (B) Representative images for mouse genotyping. (C) Representative images of Evans blue staining of mouse lungs of Gpx4^f/f^ or Gpx4^cko^, with or without AAV-shNC or AAV-shGbp2 treatment and then receiving CLP. (D) Quantification of Evans blue staining of mouse lungs (n = 8). (E) Wet to dry weight ratio of mouse lungs (n = 6). (F) Representative images of HE staining of mouse lungs and lung injury score. Scale bar = 100 μm. (G) The level of Angpt2 in the serum of mice (n = 8). (H) Representative immunoblotting and quantification of barrier-associated proteins (ZO-1, VE-Cadherin and occludin) in mouse lungs (n = 3). (I, J) Representative images of immunofluorescence staining for DAPI/CD31/ZO-1 and DAPI/CD31/occludin in mouse lungs. Scale bar = 20 μm or 50 μm. Data are presented as mean ± SD. ∗*P* < 0.05, ∗∗*P* < 0.01, ∗∗∗*P* < 0.001, and ∗∗∗∗*P* < 0.0001.

Previous studies have demonstrated that GPX4 plays a crucial regulatory role in ferroptosis [8]. To further investigate the relationship between macrophagic GBP2 and endothelial cell ferroptosis in si-ALI, the Cre-LoxP recombination system was used to generate endothelial-specific Gpx4 knockout mice (Gpx4^cko^) (Fig. 7A and B). The results demonstrated that under normal conditions, endothelial-specific Gpx4 deletion had no impact on inflammation or vascular barrier function in mouse lung tissue (Supplemental Figs. 8A–D). Compared to the Gpx4^f/f^ + AAV-shNC group, Gpx4^cko^ + AAV-shNC mice exhibited increased vascular permeability and more severe lung injury induced by CLP (Fig. 7C–F). Treatment with AAV-shGbp2 in Gpx4^f/f^ mice partially alleviated the increased vascular permeability and lung injury; however, no significant improvement was observed in Gpx4^cko^ mice treated with AAV-shGbp2 (Fig. 7C–F). Additionally, after CLP surgery, circulating levels of the vascular permeability marker Angpt2 were significantly higher in the Gpx4^cko^ + AAV-shNC group compared to the Gpx4^f/f^ + AAV-shNC group (Fig. 7G). AAV-shGbp2 treatment partially reduced Angpt2 levels in Gpx4^f/f^ mice, but no noticeable improvement was observed in Gpx4^cko^ mice treated with AAV-shGbp2 (Fig. 7G). A similar trend was observed in the levels of barrier-related proteins in lungs (Fig. 7H–J). These findings suggest that in si-ALI, macrophagic GBP2 promotes vascular barrier disruption and increases pulmonary vascular permeability by downregulating GPX4 in endothelial cells.

### 3.8 GBP2 and OTUD5 interaction promotes the ubiquitination and degradation of GPX4 in HPMECs

**Fig. 8:**
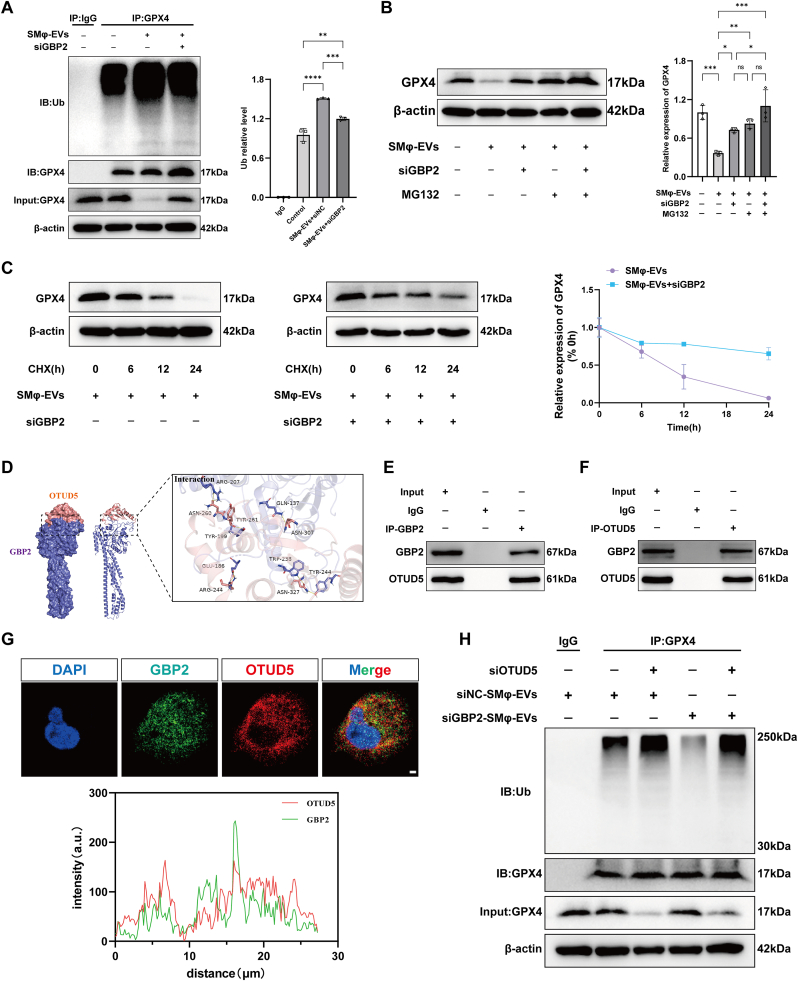
GBP2 and OTUD5 interaction promotes the ubiquitination and degradation of GPX4 in HPMECs. (A) Representative images and quantification of immunoblotting of GPX4 protein and its ubiquitination in HPMECs co-cultured with SMφ-EVs with or without silencing GBP2 (n = 3). (B) Representative images and quantification of immunoblotting of GPX4 in HPMECs in the presence or absence of MG132 pretreatment (n = 3). (C) Representative images and quantification of immunoblotting of GPX4 in HPMECs with and without cycloheximide (CHX) pretreatment (n = 3). (D) Binding model of GBP2 and OTUD5 and the residues detail of the interaction. (E, F) Representative images of co-immunoprecipitations used to assess the binding between GBP2 and OUTD5. (G) Representative images and quantification of fluorescence intensity of immunofluorescence staining for DAPI/GBP2/OTUD5 in HPMECs treated with SMφ-EVs. Scale bar = 2 μm. (H) Representative images of immunoblotting of GPX4 protein and its ubiquitination levels in HPMECs co-cultured with SMφ-EVs with or without silencing GBP2 or OTUD5. Data are presented as mean ± SD. ∗*P* < 0.05, ∗∗*P* < 0.01, ∗∗∗*P* < 0.001, and ∗∗∗∗*P* < 0.0001.

Recent studies have highlighted abnormal ubiquitination of GPX4 in several diseases related to ferroptosis [27]. To further explore how macrophage-derived EV GBP2 mediates the downregulation of GPX4 and promotes ferroptosis in vascular endothelial cells, the ubiquitination levels of GPX4 in HPMECs were examined. Our results demonstrated that GPX4 ubiquitination was significantly elevated in HPMECs co-cultured with SMφ-EVs (Fig. 8A). Reducing GBP2 levels in SMφ-EVs diminished this effect (Fig. 8A). Additionally, treatment with the proteasome inhibitor MG132 restored GPX4 protein levels in HPMECs co-cultured with SMφ-EVs, indicating that the reduction of GPX4 is associated with proteasome-dependent degradation (Fig. 8B). Furthermore, experiments using the protein synthesis inhibitor cycloheximide (CHX) showed that GBP2-induced GPX4 reduction occurs independently of new protein synthesis (Fig. 8C).

To investigate the specific mechanism of GBP2-induced the elevation of GPX4 ubiquitination, co-immunoprecipitation was used to examine whether GBP2 interacts with GPX4. The results showed no direct interaction between GBP2 and GPX4 (Supplemental Figs. 9A and B), suggesting that GBP2 regulates the ubiquitination level of GPX4 through intermediate molecules. Previous studies have identified OTUD5 as a key deubiquitinase regulating GPX4 ubiquitination [28]. Protein-protein docking analysis was performed to predicted the interaction between GBP2 and OTUD5, which revealed a binding energy of −67.42 kcal/mol, indicating a significant probability of interaction (Fig. 8D). After co-culturing SMφ-EVs with HPMECs, co-immunoprecipitation confirmed the direct binding of GBP2 to OTUD5 in HPMECs. Immunofluorescence staining further validated this interaction and indicated that it primarily occurs in the cytoplasm (Fig. 8E–G). Silencing OTUD5 increased GPX4 ubiquitination in HPMECs co-cultured with siGBP2-SMφ-EVs (Fig. 8H). These findings suggest that during si-ALI, macrophage-derived EV GBP2 promotes the ubiquitination and degradation of GPX4 by directly interacting with OTUD5 in endothelial cells.

### 3.9 Plantainoside D facilitates the dissociation of GBP2 from OTUD5 and inhibits GPX4 ubiquitination

**Fig. 9:**
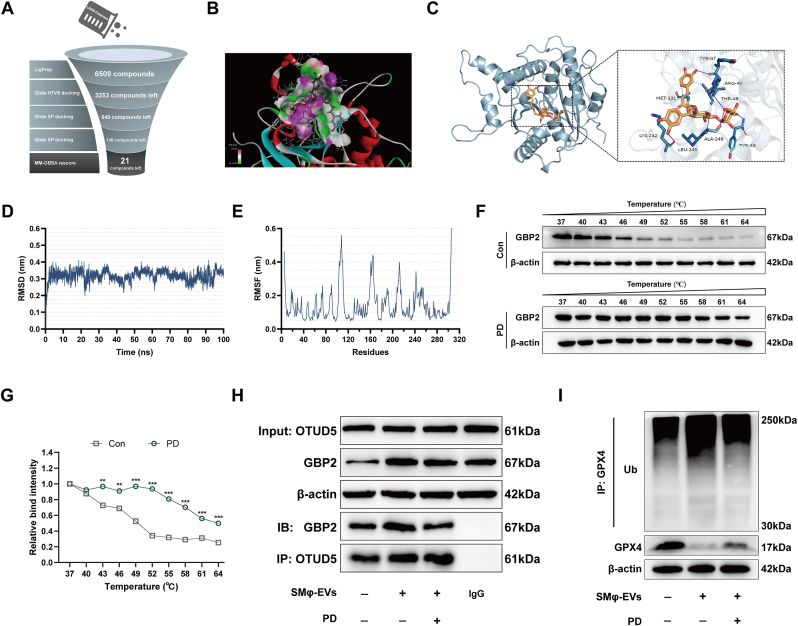
Plantainoside D facilitates the dissociation of GBP2 from OTUD5 and inhibits GPX4 ubiquitination. (A) Overview of the compound screening scheme. (B, C) The representative docking complex of GBP2 and Plantainoside D (PD). (D, E) Root mean square deviation (RMSD) and root mean square fluctuation (RMSF) values of the GBP2-PD docking complex. (F, G) Cellular thermal shift assay between PD and GBP2 (F) and the quantification (G) (n = 3). (H) Representative immunoblot images of GBP2 and OTUD5, along with co-immunoprecipitation images assessing the binding between GBP2 and OTUD5. (I) Representative images of immunoblotting of GPX4 protein and its ubiquitination levels in HPMECs co-cultured with SMφ-EVs, with or without PD treatment. Data are presented as mean ± SD. ∗*P* < 0.05, ∗∗*P* < 0.01, ∗∗∗*P* < 0.001, and ∗∗∗∗*P* < 0.0001.

To identify strategies for regulating GBP2 to improve si-ALI, the high-throughput screening of natural small molecules was conducted targeting GBP2 protein (Fig. 9A and Supplemental Table 4). Based on the screening results and the biological properties of the compounds, Plantainoside D (PD) was selected for further investigation. Molecular docking analysis revealed that PD binds to GBP2 with a binding energy of −70.72 kcal/mol (Fig. 9B and C). Molecular dynamics simulations were then used to evaluate the stability of the PD-GBP2 interaction. The result of RMSD showed that the PD-GBP2 complex reached a stable state after nearly 5 ns (Fig. 9D). Notably, residues around positions 40, 120, and 240, which interact with PD, maintained low RMSF values, indicating potent binding (Fig. 9E). Compared to the control group, the PD group exhibited increased protease resistance of GBP2, with a slower degradation rate (Fig. 9F and G). These findings confirmed the binding interaction between PD and GBP2. Furthermore, a CCK-8 assay was performed to assess the effect of different PD concentrations on HPMEC viability. The results showed that PD exhibited cytotoxicity at 20 μM, while 5–10 μM PD dose-dependently reversed SMφ-EVs-induced viability suppression, with 10 μM exerting the most pronounced protective effect (Supplemental Figs. 10A and B). Western blot analysis revealed that 10 μM PD significantly alleviated SMφ-EVs-induced downregulation of GPX4 and the loss of barrier-related proteins, with effects comparable to those of the ferroptosis inhibitor Fer-1. Notably, the combination of 10 μM PD and Fer-1 produced a synergistic therapeutic effect (Supplemental Figs. 10C and D).

The results of Western blot showed that PD did not significantly alter the levels of OTUD5 and GBP2 (Fig. 9H). However, the interaction between GBP2 and OTUD5 in HPMECs was partially inhibited by PD (Fig. 9H). Furthermore, PD treatment reduced the elevated level of GPX4 ubiquitination caused by SMφ-EVs (Fig. 9I). GBP2 is recognized as a GTP hydrolase [29]. To evaluate whether the interaction between PD and GBP2 influences GBP2 activity, the GTPase activity of purified GBP2 was assessed in the presence of increasing concentrations of PD (0–20 μM). GTPase activity was quantified by measuring the release of free phosphate generated through GTP hydrolysis. The results demonstrated that PD did not significantly affect GBP2 activity (Supplemental Figs. 10E–G). The results indicate that PD inhibits GPX4 ubiquitination and disrupts the interaction between GBP2 and OTUD5, independent of GBP2 activity.

### 3.10 Combined treatment of GBP2 blockade and PD showed additive pulmonary-protective effects in CLP mice

**Fig. 10:**
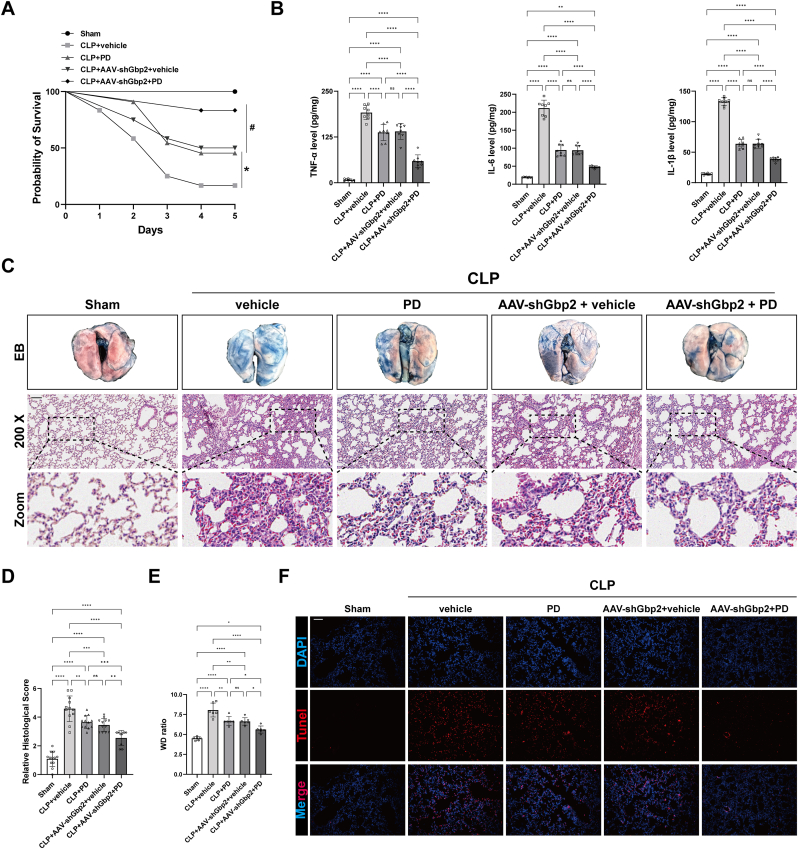
Combined treatment of GBP2 blockade and PD showed additive pulmonary-protective effects in CLP mice. (A) Survival rate of mice treated with or without CLP following administration of AAV-shGbp2 or PD treatment (n = 12). (B) Quantification of IL-6, TNF-α and IL-1β in mouse lungs (n = 8). (C) Representative images of Evans blue staining and HE staining of mouse lungs. (D) The lung injury score of mice. (E) Wet to dry weight ratio of mouse lungs (n = 6). (F) Representative images and quantification of Tunel staining of mouse lungs. Data are presented as mean ± SD. ∗*P* < 0.05, ∗∗*P* < 0.01, ∗∗∗*P* < 0.001, and ∗∗∗∗*P* < 0.0001.

**Fig. 11:**
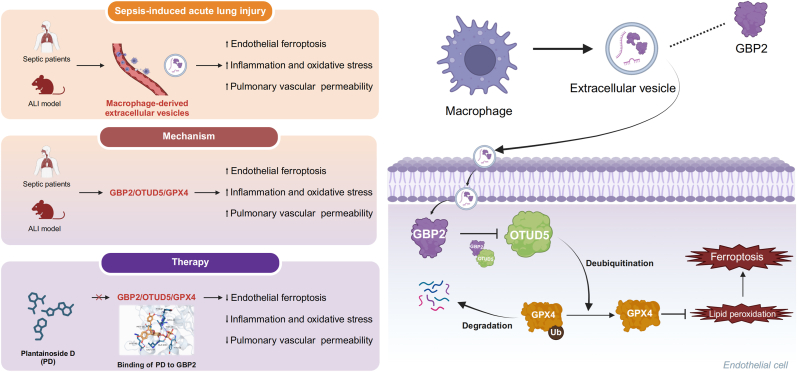
Mechanistic scheme. GBP2 is highly expressed in septic macrophages and their secreted EVs. Mechanistically, macrophagic GBP2 transfers to endothelial cells via EVs, where it interacts directly with the deubiquitinase OTUD5, promoting GPX4 ubiquitination and degradation, leading to endothelial ferroptosis. Plantainoside D binds to GBP2, inhibiting the interaction between GBP2 and OTUD5, thereby blocking the GBP2-OTUD5-GPX4 axis, preventing endothelial damage and alleviating lung injury.

Further investigation demonstrated that PD significantly improved survival rates, pulmonary vascular permeability, lung injury, and inflammation in CLP mice (Fig. 10A–F). Notably, the combination of PD and GBP2 knockdown further enhanced these improvements (Fig. 10A–F). These findings suggest a potential regulatory role of PD on si-ALI and demonstrate that GBP2 inhibition combined with PD treatment exerts an additive pulmonary-protective effect against si-ALI. Fig. 11 illustrates the mechanism by which macrophage-derived EV GBP2 mediates endothelial ferroptosis in si-ALI and the potential regulatory role of PD.

## 4 Discussion

Pulmonary vascular endothelial barrier damage is a vital feature of the acute phase of si-ALI and identifying the factors that mediate endothelial injury is critical for improving patient outcomes [30]. Macrophages contribute to the progression of si-ALI by secreting EVs to communicate with other cells [12]. However, the precise role of macrophage-derived EVs in regulating vascular endothelial cells during si-ALI remains unclear and warrants further investigation.

In this study, immunofluorescence double staining revealed increased macrophage infiltration in the lung tissue of CLP mice, with spatial proximity to vascular endothelial cells, potentially facilitating regulatory interactions. Previous studies have shown that resident lung macrophages primarily utilize the tricarboxylic acid cycle and amino acid metabolism to maintain their function, playing a critical role in pulmonary homeostasis and repair during the recovery phase of acute lung injury [31,32]. By contrast, monocyte-derived macrophages exhibit distinct metabolic profiles, characterized mainly by glycolysis and arginine metabolism, and typically display a pronounced pro-inflammatory phenotype that mediates tissue damage under pathological conditions [32]. Moreover, studies have reported that during si-ALI, the number of resident macrophages remains stable, whereas monocyte-derived macrophages progressively increase within 72 h of lung injury [31]. Therefore, we propose that the increased macrophages observed in the lung tissue of CLP mice in this study are predominantly pro-inflammatory monocyte-derived macrophages.

Previous research has shown that EVs from pro-inflammatory macrophages disrupt mitochondrial function in traumatic spinal cord injury and induce endothelial-mesenchymal transition in endothelial cells [13]. Similarly, in myocardial infarction, pro-inflammatory macrophages release EVs that promote endothelial cell death and inhibit angiogenesis [14]. These findings imply that pro-inflammatory macrophages contribute to endothelial cell damage. In the early stage of si-ALI, macrophages are also primarily pro-inflammatory [33]. These studies propose the possibility that macrophages contribute to endothelial barrier damage in the early stages of sepsis. In this study, we utilized the monocyte-derived macrophage cell line THP-1 and observed significant morphological changes and increased inflammatory cytokine expression following LPS treatment. These findings align with previous reports describing the strong pro-inflammatory properties of monocyte-derived macrophages under pathological conditions [32]. Additionally, co-culturing HPMECs with SMφ-conditioned medium led to increased endothelial cell death, accompanied by a marked reduction in ZO-1, occludin, and VE-Cadherin levels, indicating endothelial barrier damage. Our findings are partially consistent with Shen's [34], as both observed that conditioned medium from LPS-treated macrophages induced endothelial cell injury. However, Shen's study did not explore the mechanisms underlying macrophage-conditioned medium-induced endothelial injury. The SMφ-conditioned medium comprises various substances, including inflammatory cytokines. While previous studies highlighted the role of inflammatory factors in promoting endothelial disruption [[35], [36], [37]], anti-inflammatory therapies have not demonstrated optimal therapeutic efficacy for si-ALI [38], suggesting the involvement of additional factors mediating endothelial damage.

EV-mediated intercellular signaling has garnered significant attention in recent years, with macrophages recognized as a major source of EVs [39]. Our results demonstrate that pretreating SMφ with the EV synthesis/secretion inhibitor GW4869 significantly diminishes the conditioned medium's capacity to induce HPMEC injury, highlighting the pivotal role of EVs in this process. However, GW4869 did not entirely prevent the injury, possibly due to its inability to fully inhibit inflammatory cytokine secretion by SMφ. These findings indicate that both EVs and inflammatory cytokines contribute to endothelial cell injury. Further research is needed to delineate their specific roles in endothelial damage, elucidate their synergistic mechanisms, and establish a theoretical foundation for targeted endothelial protection. Additionally, we observed a time-dependent decrease in the viability of HPMECs co-cultured with SMφ-EVs, accompanied by endothelial barrier disruption after 24 h of co-culture. This result aligns partially with Ge's findings [13]. Although Ge's study focused on macrophage-derived EVs mediating vascular endothelial injury under spinal cord injury conditions, the macrophages were stimulated with LPS, consistent with the treatment used in our experiment, which may explain the similarity in findings.

Ferroptosis is characterized by lipid peroxidation and abnormal iron metabolism [40]. The balance between the oxidative system, which includes iron ions, the Fenton reaction, and ROS, and the antioxidant system, including GPX4, GSH, the Xc− system, and other newly discovered pathways, maintains cellular and systemic homeostasis [41]. During infection and inflammation, this balance is disrupted, leading to ferroptosis [42]. Additionally, studies suggest that the molecular processes underlying ferroptosis are intricately connected to the regulatory networks of other forms of programmed cell death. These forms of cell death interact synergistically, collectively exacerbating tissue damage. This underscores the pivotal role of ferroptosis in tissue injury [43,44]. In this study, we found that co-culturing HPMECs with SMφ-EVs significantly decreased the levels of GSH, SLC7A11, and GPX4, while GSSG, MDA, and lipid peroxides increased, indicating that SMφ-EVs disrupts the redox balance in endothelial cells. Treatment with the ferroptosis inhibitor Fer-1 reversed these changes, confirming that SMφ-EVs-mediated endothelial cell injury is partially associated with ferroptosis. To explore the in vivo effects of macrophage-derived EVs under septic conditions, RAW264.7 cells were treated with LPS and enriched the EVs (mEVs) before administering them intratracheally to mice. Our results revealed that mEVs induced ferroptosis in lung tissue, disrupted the endothelial barrier, and exacerbated lung injury in both sham and CLP mice. Gong et al. demonstrated that macrophage-derived EVs mediate necroptosis of alveolar epithelial cells through aminopeptidase N during sepsis [17]. Wang et al. reported that macrophage-derived EVs carrying the tRNA-derived fragment tRF-22-8BWS7K092 induce ferroptosis in alveolar epithelial cells, contributing to the pathogenesis of acute lung injury [12]. Furthermore, Ye et al. identified macrophages as the primary EV-secreting cells in the early stages of acute lung injury, with their EVs activating neutrophils and exacerbating pulmonary inflammation [45]. These studies suggest that macrophage-derived EVs interact with various cell types and influence lung injury outcomes. To explore whether EVs affect the pulmonary vascular endothelial barrier, we administered fluorescently labeled EVs intratracheally to mice and observed that vascular endothelial cells absorbed the EVs. Based on our in vivo and in vitro findings, we propose that mEV-induced lung injury in mice is at least partially attributed to ferroptosis in vascular endothelial cells, leading to endothelial barrier disruption. This mechanism may further contribute to the pathogenesis of si-ALI. Future studies should employ more advanced methods to refine the observation and analysis of EV uptake by specific cells, thereby improving our understanding of their impact on endothelial cells in si-ALI. Additionally, further research should focus on elucidating the distinct mechanisms through which macrophage-derived EVs influence different cell types, representing a key avenue for future investigation.

This study aims to investigate novel regulatory mechanisms of ferroptosis. Proteomic analysis, bioinformatics screening, and literature review were performed to identify key proteins closely associated with pulmonary vascular barrier damage, ultimately identifying GBP2 as a highly connected candidate for further investigation. GBP2, a member of the interferon-γ-inducible guanylate-binding protein family, has a molecular weight of 67 kDa, and its encoding gene is located on human chromosome 1 [46]. While most studies on GBP2 have focused on its role in oncology, particularly in promoting tumor proliferation and invasion [47], recent research has uncovered its significant immunoregulatory functions [48]. GBP2 is expressed in macrophages, yet it is not a secretory protein. Instead, it facilitates intercellular signaling through the EV-mediated pathway. Wang et al. reported the presence of GBP2 in EVs secreted by LPS-stimulated RAW264.7 cells, aligning with our sequencing data that indicate similar expression patterns in both human and mouse macrophagic EVs [49]. Further studies have demonstrated that GBP2 is a critical regulator of macrophage function and is closely associated with pyroptosis. GBP2-deficient macrophages show diminished pyroptosis and impaired secretion of IL-1β and IL-18 when exposed to LPS or bacterial outer membrane vesicles [50]. However, no studies have explored the relationship between GBP2 and endothelial cells during si-ALI. Our study demonstrates that GBP2 is highly expressed in macrophage-derived EVs during sepsis and in the peripheral blood mononuclear cells of sepsis patients. Moreover, the expression of GBP2 in peripheral blood EVs is positively correlated with vascular barrier damage markers, D-dimer, and SOFA scores, both of which are established indicators of sepsis severity [51,52]. These findings suggest that GBP2 is closely linked to endothelial integrity and the severity of sepsis.

Based on the differences between pulmonary resident macrophages and monocyte-derived macrophages, as well as the observed differential GBP2 expression, we propose that the increased GBP2 in lung tissue under si-ALI conditions primarily originates from monocyte-derived macrophages. However, the expression and functional role of GBP2 in pulmonary resident macrophages remains unclear, representing a promising area for further investigation. This study confirmed that GBP2 is a critical driver of endothelial ferroptosis mediated by SMφ-EVs using RNAi and plasmid transfection. Advanced approaches, such as CRISPR-Cas9-mediated GBP2 knockout, could be employed for further validation. Additionally, a preliminary screening of EV-associated proteins based on the protein interaction network identified CXCL2 and CXCL10 in EVs. Studies suggest that these proteins not only play a key role in inflammation regulation but may also promote ferroptosis by modulating oxidative stress [53,54], indicating their potential role as mediators of endothelial ferroptosis. Therefore, further research is needed to characterize the protein composition of SMφ-EVs, which may contribute to elucidating the pathological mechanisms of si-ALI.

Oxidative stress is a well-established driver of ferroptosis [43]. Excessive ROS accumulation induces lipid peroxidation, triggering ferroptosis, which in turn amplifies ROS production, establishing a vicious cycle [55]. Additionally, ROS impairs mitochondrial membrane potential, leading to mitochondrial dysfunction that may further promote ferroptosis by disrupting iron metabolism and lipid peroxidation [43]. Our study demonstrated that reducing GBP2 levels in SMφ-EVs markedly reduced ROS accumulation in endothelial cells and restored GPX4 expression. These findings indicate that GBP2 is a critical regulator within the ferroptosis-oxidative stress network. However, the precise mechanism by which GBP2 mediates endothelial ferroptosis remains to be elucidated.

GPX4 downregulation plays a critical role in ferroptosis during si-ALI, but the underlying mechanisms remain unclear and require further investigation. Recent studies have identified abnormal GPX4 ubiquitination in various ferroptosis-related diseases. The cysteine protease inhibitor SN has been shown to recruit members of the ovarian tumor domain-containing cysteine protease superfamily, reduce GPX4 ubiquitination, lower ROS levels, and inhibit ferroptosis in gastric cancer cells [56]. Some studies suggest that targeting GPX4 ubiquitination may affect disease progression, highlighting the importance of regulating ubiquitination to maintain GPX4 stability [57,58]. However, whether GPX4 downregulation in sepsis involves abnormal ubiquitination remains unknown. Our study found that GBP2 interacts with OTUD5 in endothelial cells, leading to increased GPX4 ubiquitination. Studies have shown that GPX4 ubiquitination increases cellular susceptibility to ferroptosis and apoptosis, underscoring its critical role in regulating programmed cell death [57]. Consistently, this study found that reducing GBP2 levels in SMφ-EVs reversed the upregulation of apoptosis markers in HPMECs, possibly through GBP2-mediated GPX4 ubiquitination and degradation. However, alternative mechanisms cannot be ruled out and require further experimental validation.

OTUD5, a deubiquitinating enzyme from the OTU family [59], is composed of 571 amino acids and contains two key domains: an ovarian tumor domain and a ubiquitin-interacting motif. The ovarian tumor domain is activated by phosphorylation of serine 177 by casein kinase II, enabling it to hydrolyze ubiquitin chains on substrates or free ubiquitin chains [60]. Recent research suggests that OTUD5 removes K48-linked polyubiquitin chains from GPX4, stabilizing the protein, preventing its degradation, and inhibiting ferroptosis [28]. These findings support our conclusion that GBP2-OTUD5 interactions mediate increased GPX4 ubiquitination, though further studies are required to fully elucidate the underlying mechanism.

The study confirmed the role of the GBP2-OTUD5-GPX4 axis in inducing ferroptosis and compromising endothelial barrier integrity during si-ALI. Building on this, therapeutic strategies targeting this axis were explored. With their diverse structures and biological activities, natural small molecules offer promising avenues for drug development. PD was identified as a strong binder to GBP2. PD, an active compound in Plantago, is known for its anti-inflammatory, detoxifying, and pain-relieving properties. Previous studies have suggested its therapeutic potential in si-ALI [20], though the underlying mechanisms remain unclear. Our molecular docking, dynamics simulations, and CETSA experiments confirmed PD's strong binding affinity to GBP2. We further found that PD treatment inhibits the interaction between GBP2 and OTUD5 in HPMECs, reducing GPX4 ubiquitination. In vivo, PD administered effectively alleviated lung injury in CLP mice. Additionally, PD did not alter the GTP hydrolysis activity of GBP2. These findings suggest that the therapeutic effect of PD may result from its interaction with GBP2, leading to changes in GBP2 conformation or post-translational modifications rather than its enzymatic activity. This reduces GBP2's interaction with OTUD5 and its inhibitory effect on OTUD5. However, the possibility that PD directly influences OTUD5 activity cannot be excluded. Furthermore, the precise mechanism by which PD affects the GBP2-OTUD5 interaction remains unclear and requires further investigation. Interestingly, combining PD treatment with macrophage GBP2 knockdown produced even greater improvements in lung injury. We propose two potential explanations: (1) PD may enhance the suppressive effects of GBP2 knockdown; (2) the GBP2-OTUD5-GPX4 axis may not be the sole pathway through which PD acts. Additionally, the absence of a recognized GBP2-specific inhibitor prevents direct comparison between PD and a GBP2 inhibitor. Future studies should address these limitations to better define the therapeutic effects of PD on si-ALI and further elucidate its underlying mechanisms. This study highlights the potential of PD to mitigate si-ALI by inhibiting GBP2-mediated ferroptosis. However, its clinical translation presents several challenges. First, the bioavailability and pharmacokinetic properties of PD remain poorly understood. Second, despite its high binding specificity to GBP2, the potential off-target effects of PD require further evaluation. Future research should refine these aspects to facilitate its clinical application.

This study is the first to explore the pathological mechanism by which macrophage-derived extracellular vesicle GBP2 promotes GPX4 ubiquitination and degradation through interaction with OTUD5 in endothelial cells, thereby increasing ferroptosis and exacerbating lung injury during si-ALI. However, several limitations exist in our research. First, our investigation was restricted to the acute exudative phase, focusing on macrophage-endothelial interactions and the function of GBP2 from macrophage-derived EVs during this phase. However, these aspects during later stages remain unexplored and warrant further investigation. Second, although this study confirmed the regulatory interaction between GBP2 and OTUD5 in endothelial cells, the possibility that GBP2 modulates ferroptosis through interactions with endothelial cell receptors cannot be entirely excluded. Further research is required to elucidate the precise mechanism and deepen understanding of the GBP2-mediated ferroptosis regulatory network. Third, the mechanisms through which PD regulates the GBP2-OTUD5-GPX4 axis were not elucidated. Finally, the expression and functional patterns of GBP2 in different macrophage subsets were not examined. Future research should aim to elucidate these mechanisms.

## 5 Conclusions

We have identified the role of the GBP2-OTUD5-GPX4 axis in the pathogenesis of sepsis-induced acute lung injury (si-ALI) and its impact on ferroptosis. Our results demonstrate that macrophage-derived extracellular vesicle GBP2 increases during si-ALI and is transferred to vascular endothelial cells, where it interacts with OTUD5 to promote the ubiquitination and degradation of GPX4. Knocking down GBP2 in macrophages significantly improves si-ALI. Furthermore, PD exhibits a potent binding affinity to GBP2 and effectively mitigates si-ALI, amplifying the protective effects of GBP2 inhibition. These findings enhance our understanding of si-ALI and present novel therapeutic possibilities.

## CRediT authorship contribution statement

**Zhixi Li:** Writing – original draft, Project administration, Methodology, Conceptualization. **Yue Bu:** Resources, Methodology. **Cheng Wang:** Investigation, Formal analysis. **Yongjing Yu:** Visualization, Validation. **Lei Han:** Methodology, Investigation. **Chang Liu:** Methodology, Investigation. **Guangmin Chen:** Resources. **Chenglong Li:** Resources. **Yan Zhang:** Software. **Hang Cao:** Software. **Zhaoxue Ma:** Software. **Ziyong Yue:** Writing – review & editing, Supervision, Funding acquisition.

## Funding

This work was supported by the China Postdoctoral Science Foundation under Grant Number 2024MD763969 and the Second Affiliated Hospital of Harbin Medical University.

## Declaration of competing interest

The authors declare that they have no known competing financial interests or personal relationships that could have appeared to influence the work reported in this paper.

## Data Availability

The data utilized and examined in the present investigation can be obtained from the corresponding author upon a reasonable inquiry.
