# Supplementary material for: Extracellular vesicle-packaged GBP2 from macrophages aggravates sepsis-induced acute lung injury by promoting ferroptosis in pulmonary vascular endothelial cells
Source: Redox Biol. 2025 Mar 25;82:103614. doi: 10.1016/j.redox.2025.103614 (PMC11994402; doi:10.1016/j.redox.2025.103614)
Supplement: Multimedia component 2 [file mmc2.docx]

**Supplemental figures and tables**

**Extracellular vesicle-packaged GBP2 from macrophages aggravates sepsis-induced acute lung injury by promoting ferroptosis in pulmonary vascular endothelial cells**

Zhixi Li ^a, b, c, d^, Yue Bu ^a, b, e^, Cheng Wang ^f^, Yongjing Yu ^a, b, c, d^, Lei Han ^a, b^, Chang Liu ^b, c, d, g^, Guangmin Chen ^h^, Chenglong Li ^i^, Yan Zhang ^a, b^, Hang Cao ^a, b^, Zhaoxue Ma ^a, b^ and Ziyong Yue ^a, b, *^

^a^ Department of Anesthesiology, Second Affiliated Hospital of Harbin Medical University, Harbin 150001, PR China

^b^ The Key Laboratory of Anesthesiology and Intensive Care Research of Heilongjiang Province, Harbin 150001, PR China

^c^ The Key Laboratory of Myocardial Ischemia Organization, Chinese Ministry of Education, Harbin 150001, PR China

^d^ State Key Laboratory of Frigid Zone Cardiovascular Diseases, Harbin 150001, PR China

^e^ Department of Pain Medicine, Second Affiliated Hospital of Harbin Medical University, Harbin 150001, PR China

^f^ Department of Environmental Hygiene, School of Public Health, Harbin Medical University, Harbin 150081, PR China

^g^ Department of Anesthesiology, Harbin Medical University Cancer Hospital, Harbin 150081, PR China

^h^ Department of Anesthesiology, First Affiliated Hospital of Harbin Medical University, 199 Dazhi Road, Harbin 150001, PR China

^i^ Department of Anesthesiology, Fourth Affiliated Hospital of Harbin Medical University, 37 Yiyuan Road, Harbin 150001, PR China

**^*^ Corresponding author**

Department of Anesthesiology, Second Affiliated Hospital of Harbin Medical University, Harbin 150001, PR China.

*E-mail address:* yueziyong@126.com

*Phone number:* +86 13936138861

**Supplemental Table 1. Primer pairs for Quantitative real-time PCR**

| Human TNF-α-F | CCAGGGACCTCTCTCTAATCA |
| --- | --- |
| Human TNF-α-R | TCAGCTTGAGGGTTTGCTAC |
| Human IL-6-F | CACTCACCTCTTCAGAACGAAT |
| Human IL-6-R | GCTGCTTTCACACATGTTACTC |
| Human IL-1β-F | CAGAAGTACCTGAGCTCGCC |
| Human IL-1β-R | AGATTCGTAGCTGGATGCCG |
| Human GBP2-F | CTGCTAAACTTCGGGAACAGGA |
| Human GBP2-R | TGCACTTCCCAGACGATTTGTT |
| Mouse Gbp2-F | CTGCACTATGTGACGGAGCTA |
| Mouse Gbp2-R | GAGTCCACACAAAGGTTGGAAA |
| Human GAPDH-F | GTGCCGCCTGGAGAAAC |
| Human GAPDH-R | AAGGTGGAAGAGTGGGAGT |
| Mouse Gapdh-F | AGGTCGGTGTGAACGGATTTG |
| Mouse Gapdh-R | TGTAGACCATGTAGTTGAGGTCA |

**Supplemental Table 2. Small interfering RNA sequences**

| **Name** | **Sense** | **Anti-sense** |
| --- | --- | --- |
| mGbp2-1 | GACGAACAGAAACAGUUAUUAdTdT | UAAUAACUGUUUCUGUUCGUCdTdT |
| mGbp2-2 | GCGACUGUGCAUCAGGAAAUUdTdT | AAUUUCCUGAUGCACAGUCGCdTdT |
| mGbp2-3 | GAUGUUGUUGAAACACUUCUAdTdT | UAGAAGUGUUUCAACAACAUCdTdT |
| Negative-control | UUCUCCGAACGUGUCACGUdTdT | ACGUGACACGUUCGGAGAAdTdT |
| hGBP2-1 | GCUUUCGCUAAAGCUAAGAAAdTdT | UUUCUUAGCUUUAGCGAAAGCdTdT |
| hGBP2-2 | CCCUUUAGAAGAAGAUGUCAAdTdT | UUGACAUCUUCUUCUAAAGGGdTAT |
| hGBP2-3 | GCCAAUAUGUAACAUACUCUAdTdT | UAGAGUAUGUUACAUAUUGGCdTdT |
| Negative-control | UUCUCCGAACCUGUCACGUdTdT | ACGUGACACGUUCGGAGAAdTdT |

| **Supplemental Table 3. Demographic and clinical characteristics of septic patients with high or low expression of GBP2.** | | | |
| --- | --- | --- | --- |
| Characteristics | Low expression | High expression | *P* value |
|  | （n = 28） | （n = 28） |  |
| Age, y | 51.4 ± 10.4 | 58.7 ± 12.8 | 0.714 |
| Gender, m/f | 17/11 | 13/15 | 0.284 |
| SOFA score | 7 (5-9.25) | 10 (8-12) | < 0.001 |
| Hypercholesterolemia (n) | 13 | 12 | 0.788 |
| Hypertension (n) | 8 | 12 | 0.265 |
| Diabetes mellitus (n) | 7 | 7 | 1 |
| D-dimer (μg/L) | 481(285-807.5) | 895 (485.75-1309.2) | 0.002 |
| Fibrinogen (g/L) | 3.02 ± 0.69 | 3.24 ± 0.47 | 0.168 |
| APTT (sec) | 31.3 ± 4.56 | 30.66 ± 5.27 | 0.629 |
| PT (sec) | 14.42 ± 3.9 | 13.08 ± 3.92 | 0.205 |

SOFA, sequential organ failure assessment; APTT, activated partial thromboplastin time; PT, prothrombin time. *P* < 0.05 was considered statistically significant.

**Supplemental Table 4. The 21 compounds targeting GBP2**

| **Titile** | **MMGBSA** **dG Bi** | **2D structure** |
| --- | --- | --- |
| TN7085 | -71.32 |  |
| T5785 | -70.9 |  |
| T5796 | -70.72 |  |
| T8164 | -70.06 |  |
| T20793 | -69.75 |  |
| TN1470 | -65.63 |  |
| T5S1526 | -65.35 |  |
| T3S1816 | -60.21 |  |
| T6S1597 | -60.15 |  |
| T6S0221 | -58.71 |  |
| T3683 | -57.85 |  |
| TN1471 | -57.22 |  |
| T5713 | -56.53 |  |
| TN7057 | -55.8 |  |
| T3375 | -55.47 |  |
| TN1516 | -53.91 |  |
| T4589 | -53.31 |  |
| T22417 | -51.76 |  |
| TN1166 | -51.6 |  |
| T5733 | -51.13 |  |
| T2S1158 | -50.66 |  |

**Supplemental Figures**

**Supplemental Fig. 1.** LPS induces a heightened pro-inflammatory response in THP-1 cells. (A) Representative bright field images of THP-1 cells after LPS treatment. Scale bar = 100 μm. (B-D) Quantification of IL-6 (B), TNF-α (C) and IL-1β (D) in THP-1 cells (n = 3-4). Data are presented as mean ± SD. ***P* < 0.01, ****P* < 0.001 and *****P* < 0.0001.

**Supplemental Fig. 2.** SMφ-derived EVs are internalized by endothelial cells, inducing lipid peroxidation and reducing mitochondrial membrane potential. (A) Representative immunofluorescence staining of Hoechst/phalloidin/PKH67 after co-culture with human pulmonary microvascular endothelial cells (HPMECs) using PKH67-labeled EVs. Scale bar = 20 μm. (B) Quantification of mitochondrial membrane potential of HPMECs (n = 3). (C) Quantitative analysis of liperfluo fluorescence intensity in HPMECs (n = 3). Data are presented as mean ± SD. **P* < 0.05, ***P* < 0.01, ****P* < 0.001, and *****P* < 0.0001.

**Supplemental Fig. 3.** Characterization and tracking of EVs derived from LPS-treated RAW264.7 cells. (A) Representative nanoparticle tracking analysis images showing the distribution and average diameter of mEVs. (B) Representative immunoblotting of mEV markers. (C) Representative immunofluorescence staining for DAPI/CD31/PKH67 in mouse lungs after intratracheal injection of PKM67-labeled EVs. Scale bar = 20 μm.

**Supplemental Fig. 4.** GBP2 is highly expressed in LPS-treated macrophages. (A) mRNA levels of GBP2 in THP-1 cells (n = 4). (B) Immunofluorescence staining image of GBP2 in THP-1 cells. Scale bar = 50 μm. (C) mRNA levels of GBP2 in RAW264.7 cells (n = 4). (D) Immunofluorescence staining image of GBP2 in RAW264.7 cells. Scale bar = 20 μm. (E) Representative images of immunofluorescence staining for DAPI/F4/80/GBP2 in lung tissue of mice with or without LPS intraperitoneal injection. Scale bar = 20 μm. (F) Representative immunoblot of GBP2 expression in lung tissues of mice with or without LPS intraperitoneal injection. (G-H) Representative immunofluorescence images of DAPI/CD31/GBP2 (G) and DAPI/CD31/GPX4 (H) staining in mouse lung tissue at different time points after CLP. Scale bar = 20 μm. Data are presented as mean ± SD. **P* < 0.05, ***P* < 0.01, ****P* < 0.001, and *****P* < 0.0001.

**Supplemental Fig. 5.** Macrophage-derived EVs overexpressing GBP2 exacerbate ferroptosis in pulmonary vascular endothelial cells during si-ALI. (A) Representative images and quantification of the ROS staining in HPMECs co-cultured with Mφ-EVs and SMφ-EVs with or without silencing GBP2 (n = 3). Scale bar = 200 μm. (B) Representative images and quantification of apoptotic markers (cleaved caspase-3 and cleaved PARP) in HPMECs (n = 3). (C) Quantification of barrier-associated proteins (ZO-1, VE-Cadherin and occludin) and ferroptosis-associated proteins GPX4 in HPMECs (n = 3). (D) Representative immunoblotting for efficiency validation after overexpressing GBP2 and RT-qPCR analysis to determine mRNA expression of GBP2 in THP-1 cells (n = 4). (E) C11-BODIPY assay evaluating the lipid ROS levels in HPMECs (n=3). (F, G) Representative images and quantification of the ROS staining in HPMECs co-cultured with Mφ-EVs and SMφ-EVs with or without overexpressing GBP2 (n = 3). Scale bar = 200 μm. (H) Representative immunoblotting and quantification of barrier-associated proteins (ZO-1, VE-Cadherin and occludin) and ferroptosis-associated proteins (SLC7A11 and GPX4) in HPMECs (n = 3). Data are presented as mean ± SD. **P* < 0.05, ***P* < 0.01, ****P* < 0.001, and *****P* < 0.0001.

**Supplemental Fig. 6.** Macrophage-derived EVs are involved in si-ALI through GBP2-induced ferroptosis. (A) Representative immunoblotting for efficiency validation after silencing GBP2 in RAW264.7 cells (n = 4). (B) Transmission electron microscopy (TEM) images of the isolated EVs from the medium of LPS-treated RAW264.7 after silencing GBP2. Scale bar = 100 nm. (C) Representative nanoparticle tracking analysis images showing the distribution and average diameter of siGbp2-mEVs. (D) Representative immunoblotting of GBP2 after overexpressing GBP2 in RAW264.7 cells (n = 4). (E) TEM images of the isolated EVs of LPS-treated RAW264.7 after overexpressing GBP2. Scale bar = 100 nm. (F) Representative nanoparticle tracking analysis images showing the distribution and average diameter of oeGbp2-mEVs. (G) Representative immunoblotting of mEV markers. (H, I) Representative images of HE staining of lung tissue and relative lung injury score of mice undergoing intratracheal instillation of mEVs and receiving or non-receiving CLP. Scale bar = 50 μm. (J, K) Representative images of Tunel and DHE staining of mouse lungs. Scale bar = 100 μm or 50 μm. (L) Representative immunoblotting and quantification of barrier-associated proteins (ZO-1, VE-Cadherin and occludin) and ferroptosis-associated proteins (SLC7A11 and GPX4) in mouse lungs (n = 3). Data are presented as mean ± SD. **P* < 0.05, ***P* < 0.01, ****P* < 0.001, and *****P* < 0.0001.

**Supplemental Fig. 7.** Validation of the biosafety and efficiency of macrophage-specific knockdown of Gbp2 in mice. (A) Representative images of DAPI/F4/80/GBP2 immunofluorescence staining in mouse lungs. Scale bar = 50 μm. (B) Representative HE-staining images of lung tissues from mice treated with AAV-NC or AAV-Gbp2. Scale bar = 50 μm.

**Supplemental Fig. 8.** The impact of endothelial-specific Gpx4 deletion on mouse lung tissue. (A) Representative HE-staining images of lung tissues from Gpx4^f/f^ and Gpx4^cko^ mice. Scale bar = 100 μm. (B) Quantification of IL-6, TNF-α and IL-1β in mouse lungs (n = 8). (C) Representative images of Evans blue staining of mouse lungs of Gpx4^f/f^ and Gpx4^cko^ mice. (D) Representative immunoblotting of barrier-associated proteins (ZO-1, VE-Cadherin and occludin) in mouse lungs (n = 3). Data are presented as mean ± SD. **P* < 0.05, ***P* < 0.01, ****P* < 0.001, and *****P* < 0.0001.

**Supplemental Fig. 9.** GBP2 does not directly interact with GPX4. (A, B) Representative images of co-immunoprecipitations used to assess the binding between GBP2 and GPX4.

**Supplemental Fig. 10.** Plantainoside D inhibits SMφ-EVs-induced ferroptosis independently of its GTPase activity. (A) The effect of increasing Plantainoside D (PD) concentrations on cell viability was assessed using the CCK-8 assay (n = 5). (B) HPMECs were treated with either the vehicle alone or varying concentrations of PD, followed by exposure to Mφ-EVs or SMφ-EVs for 24 h, and cell viability was assessed using the CCK-8 assay (n = 5). (C-D) Representative images (C) and quantification (D) of barrier and ferroptosis markers (ZO-1, VE-Cadherin, occludin and GPX4) in HPMECs (n = 3). (E) GTPase activity measured with increasing concentrations of PD. (F) GTPase activity measured with increasing concentrations of GBP2. (C) GBP2 (100nM) incubated with increasing concentrations of PD.
